# Supplementary figures and images for: Mycobacterial metallophosphatase MmpE acts as a nucleomodulin to regulate host gene expression and promote intracellular survival
Source: eLife. 2026 Mar 18;14:RP108037. doi: 10.7554/eLife.108037 (PMC12999176; doi:10.7554/eLife.108037)

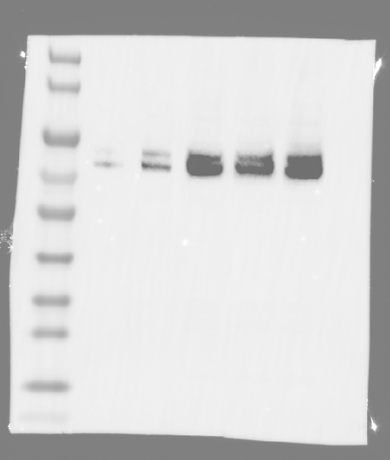

Supplement: Figure 1—source data 1. [file elife-108037-fig1-data1.zip › eLife-108037R1-Figure 1-sourse data/Figure 1B-GFP-1.tif]

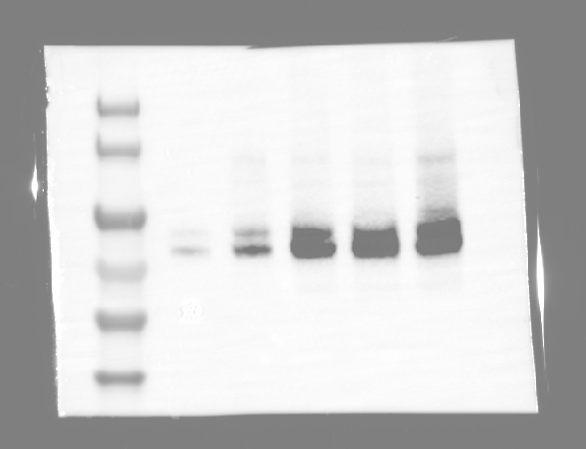

Supplement: Figure 1—source data 1. [file elife-108037-fig1-data1.zip › eLife-108037R1-Figure 1-sourse data/Figure 1B-GFP-2.tif]

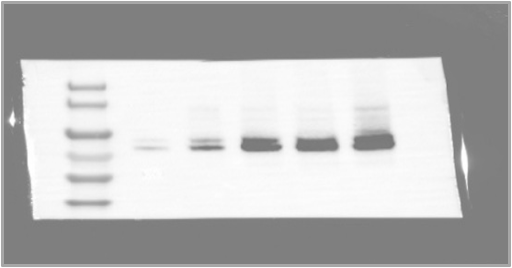

Supplement: Figure 1—source data 1. [file elife-108037-fig1-data1.zip › eLife-108037R1-Figure 1-sourse data/Figure 1B-GFP-3.tif]

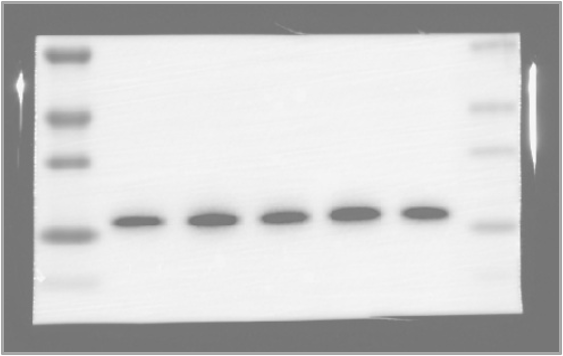

Supplement: Figure 1—source data 1. [file elife-108037-fig1-data1.zip › eLife-108037R1-Figure 1-sourse data/Figure 1B-H3.tif]

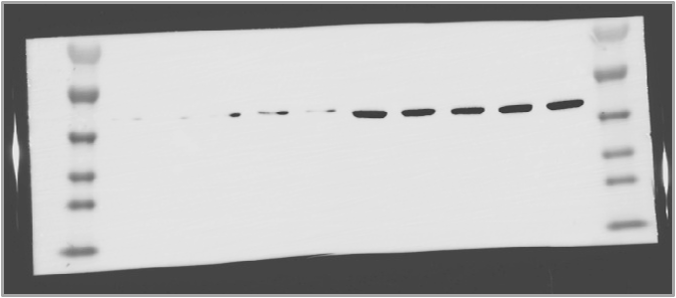

Supplement: Figure 1—source data 1. [file elife-108037-fig1-data1.zip › eLife-108037R1-Figure 1-sourse data/Figure 1F-b-actin.tif]

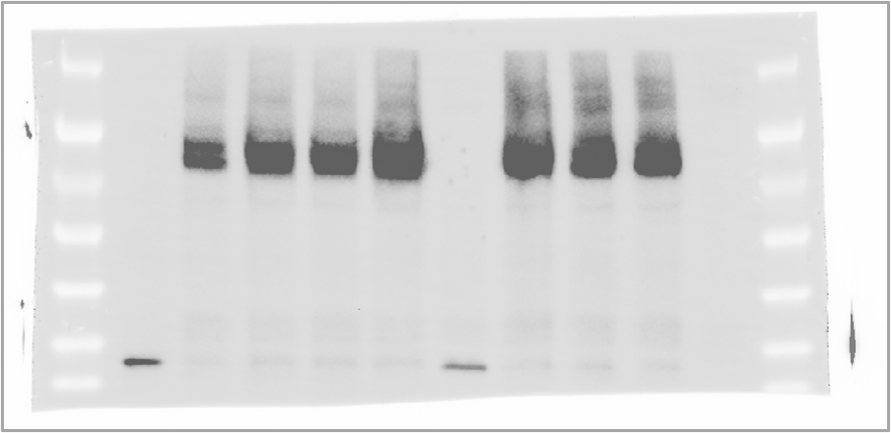

Supplement: Figure 1—source data 1. [file elife-108037-fig1-data1.zip › eLife-108037R1-Figure 1-sourse data/Figure 1F-GFP-1.tif]

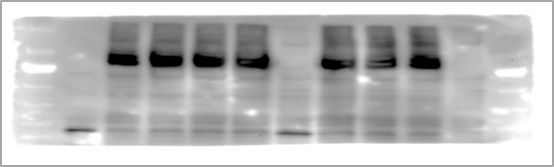

Supplement: Figure 1—source data 1. [file elife-108037-fig1-data1.zip › eLife-108037R1-Figure 1-sourse data/Figure 1F-GFP-2.tif]

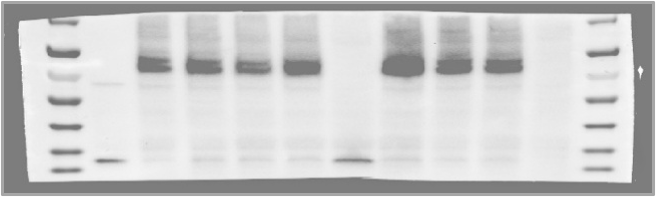

Supplement: Figure 1—source data 1. [file elife-108037-fig1-data1.zip › eLife-108037R1-Figure 1-sourse data/Figure 1F-GFP-3.tif]

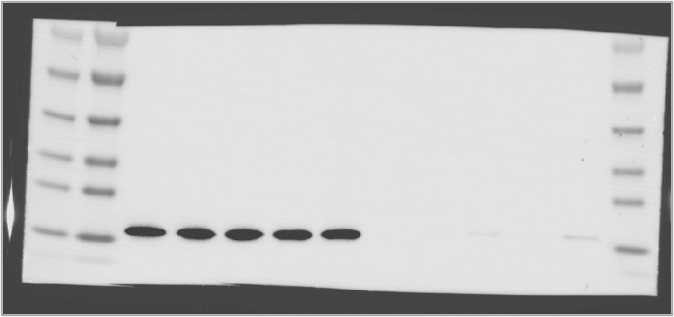

Supplement: Figure 1—source data 1. [file elife-108037-fig1-data1.zip › eLife-108037R1-Figure 1-sourse data/Figure 1F-H3.tif]

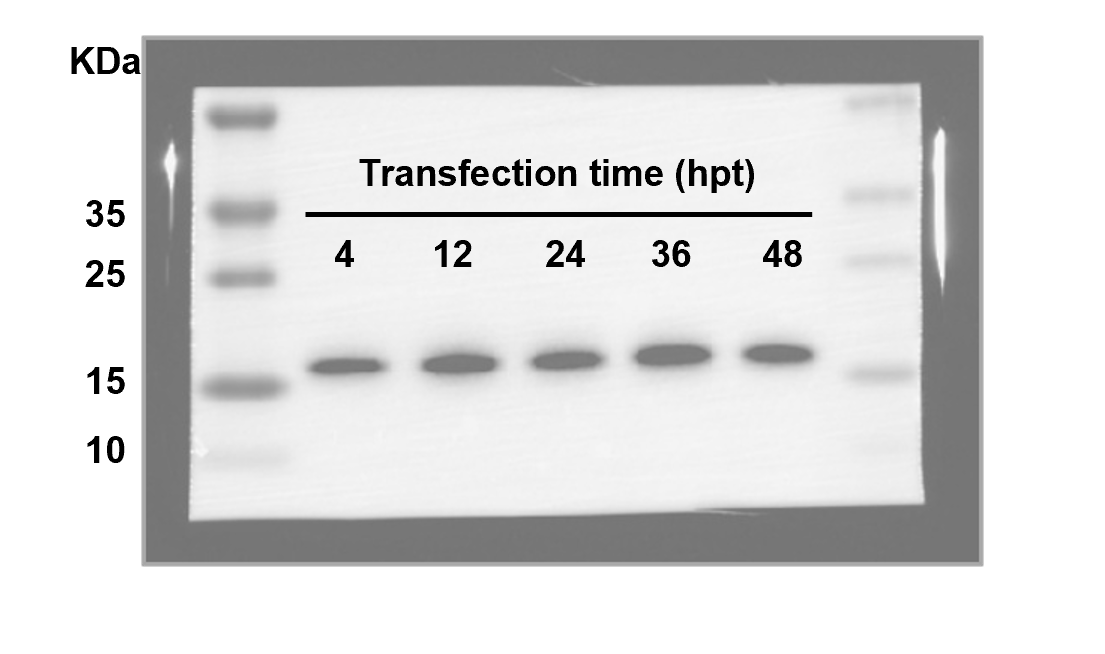

Supplement: Figure 1—source data 2. [file elife-108037-fig1-data2.zip › eLife-108037R1-Figure 1-sourse data and notes/Figure 1B-H3.tif]

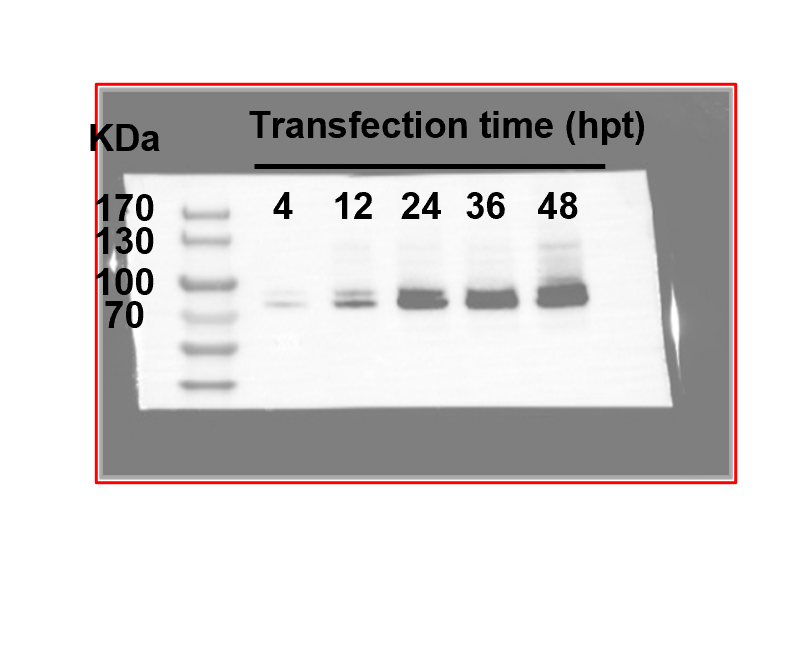

Supplement: Figure 1—source data 2. [file elife-108037-fig1-data2.zip › eLife-108037R1-Figure 1-sourse data and notes/Figure 1B-MmpE-GFP-1.tif]

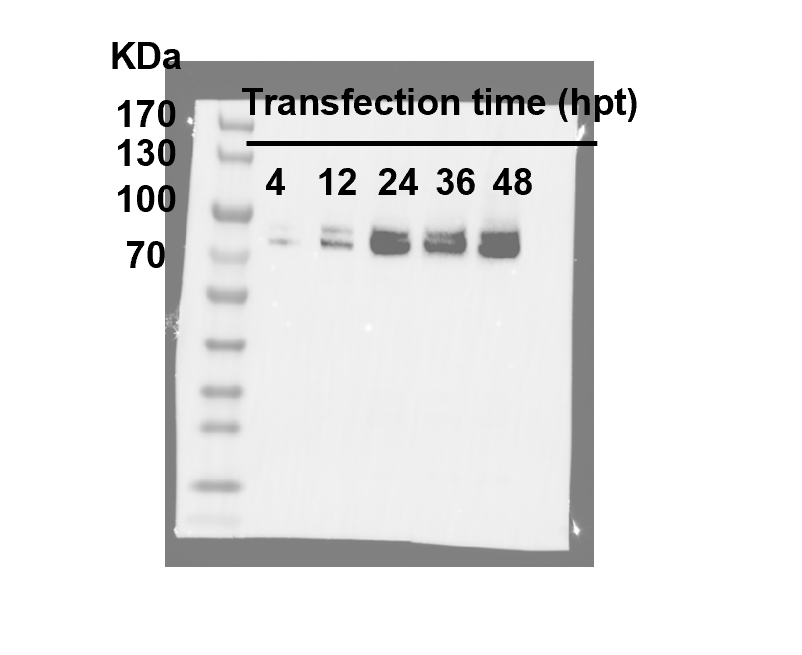

Supplement: Figure 1—source data 2. [file elife-108037-fig1-data2.zip › eLife-108037R1-Figure 1-sourse data and notes/Figure 1B-MmpE-GFP-2.tif]

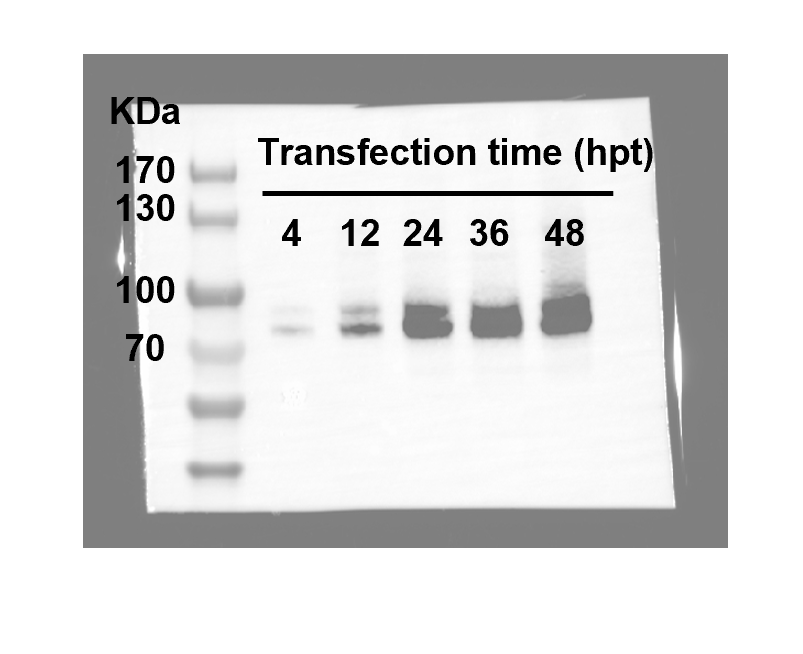

Supplement: Figure 1—source data 2. [file elife-108037-fig1-data2.zip › eLife-108037R1-Figure 1-sourse data and notes/Figure 1B-MmpE-GFP-3.tif]

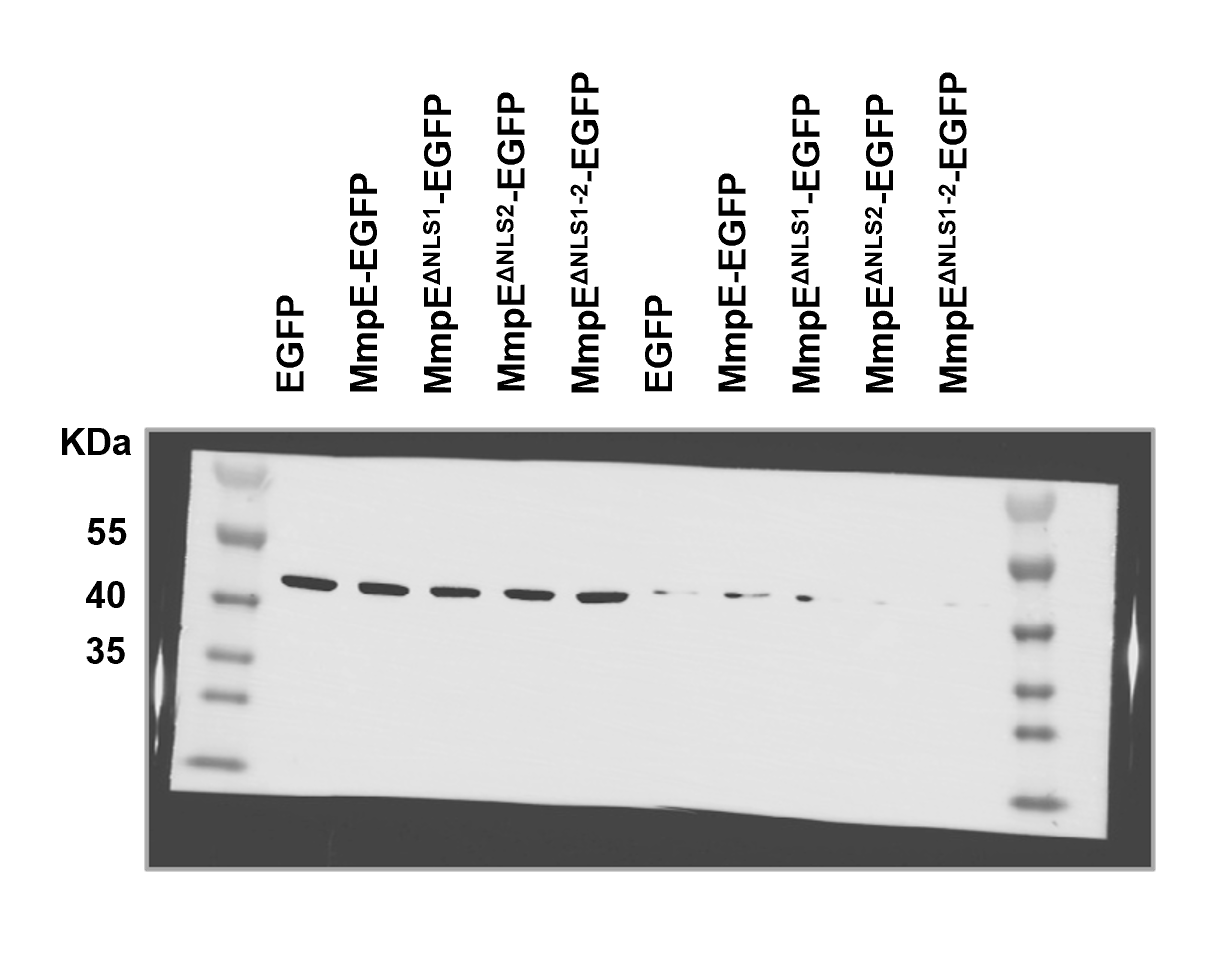

Supplement: Figure 1—source data 2. [file elife-108037-fig1-data2.zip › eLife-108037R1-Figure 1-sourse data and notes/Figure 1F-b-actin.tif]

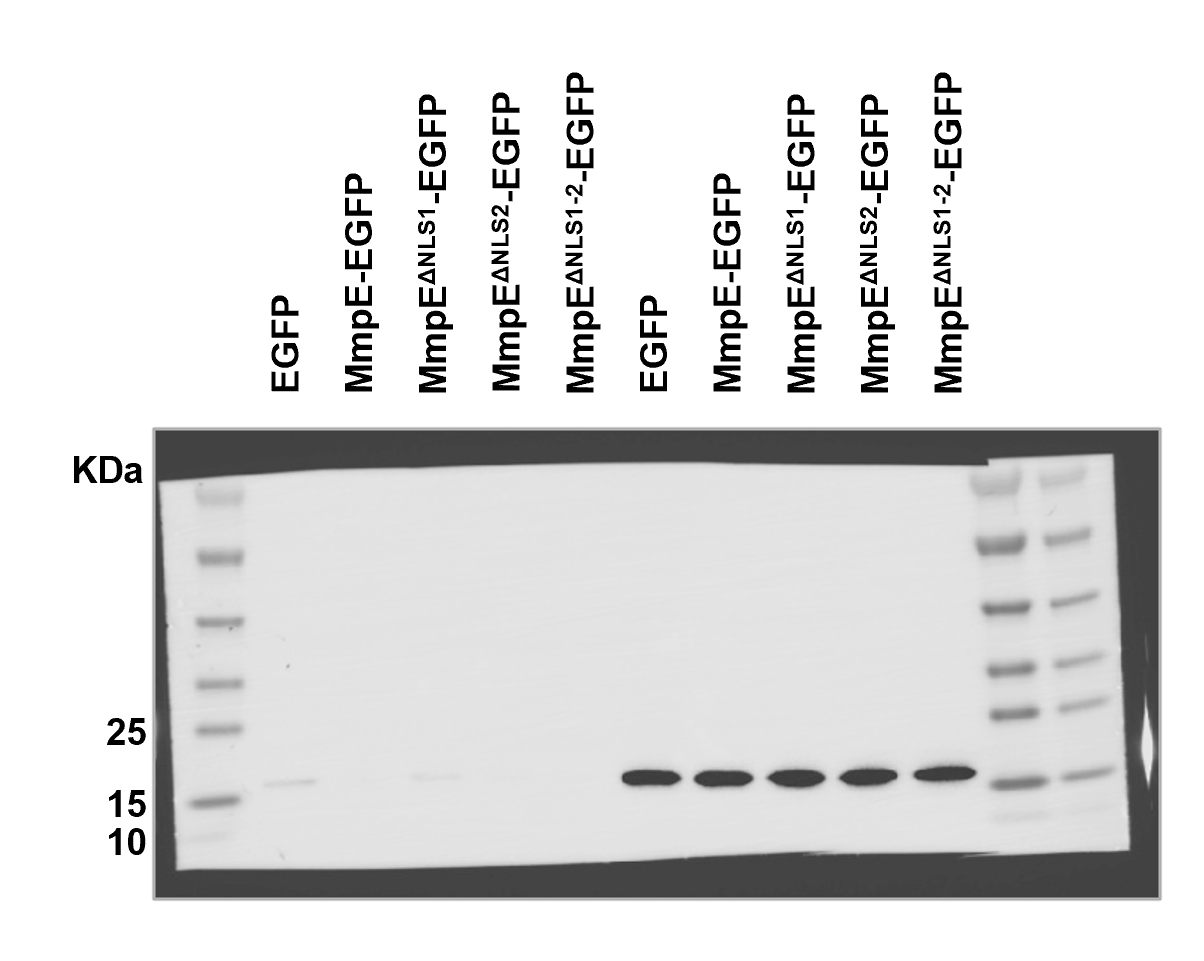

Supplement: Figure 1—source data 2. [file elife-108037-fig1-data2.zip › eLife-108037R1-Figure 1-sourse data and notes/Figure 1F-H3.tif]

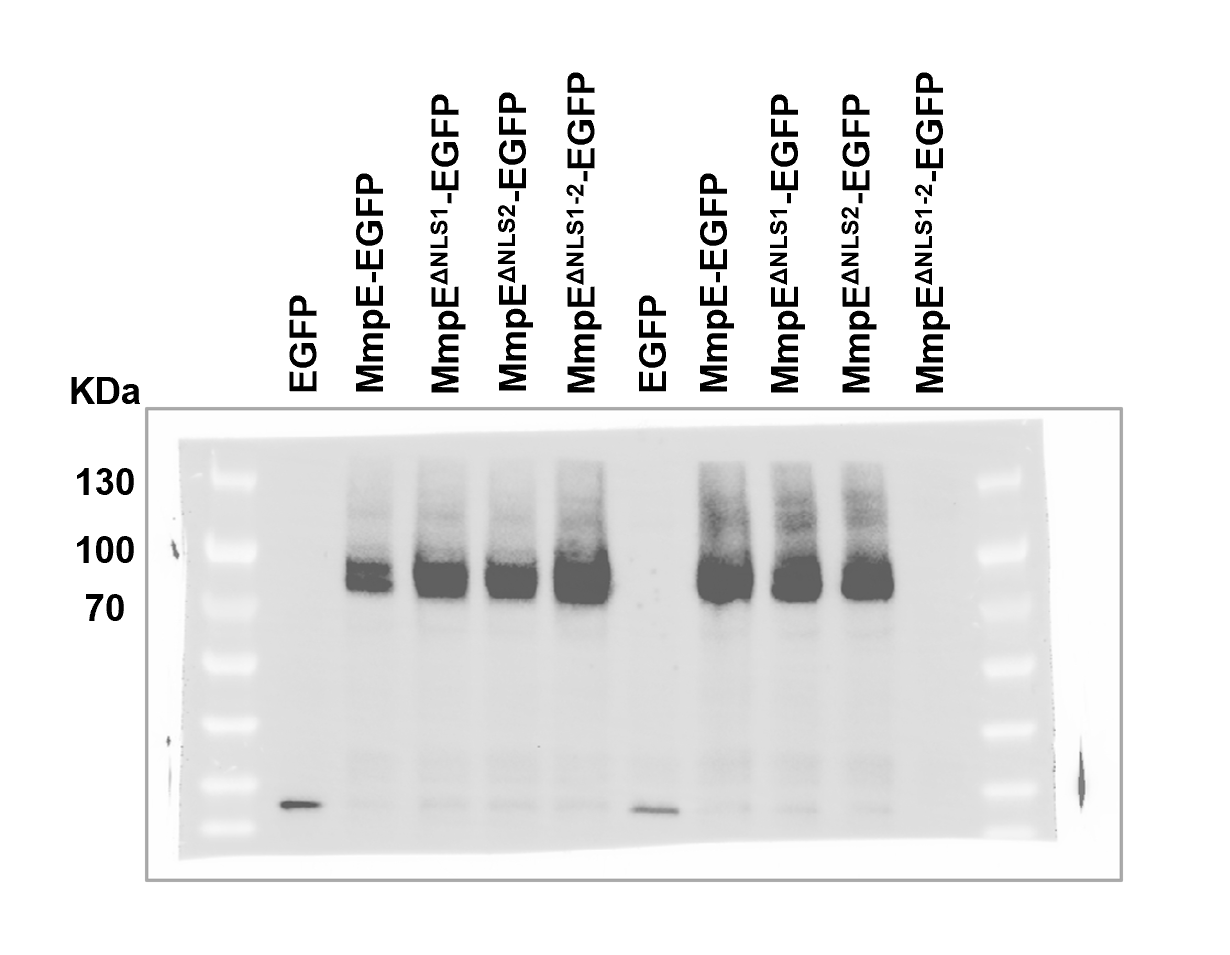

Supplement: Figure 1—source data 2. [file elife-108037-fig1-data2.zip › eLife-108037R1-Figure 1-sourse data and notes/Figure 1F-MmpE-GFP-1.tif]

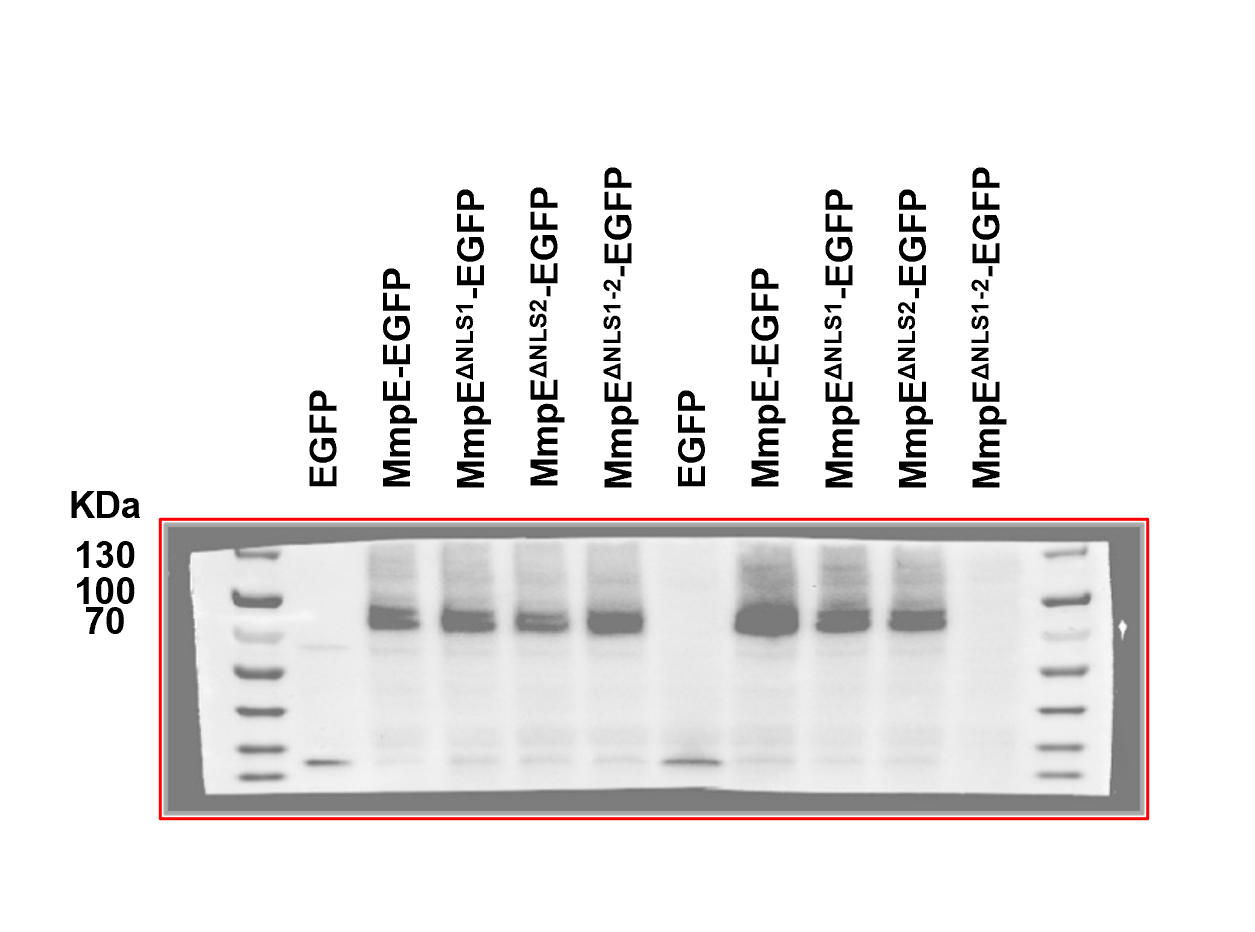

Supplement: Figure 1—source data 2. [file elife-108037-fig1-data2.zip › eLife-108037R1-Figure 1-sourse data and notes/Figure 1F-MmpE-GFP-2.tif]

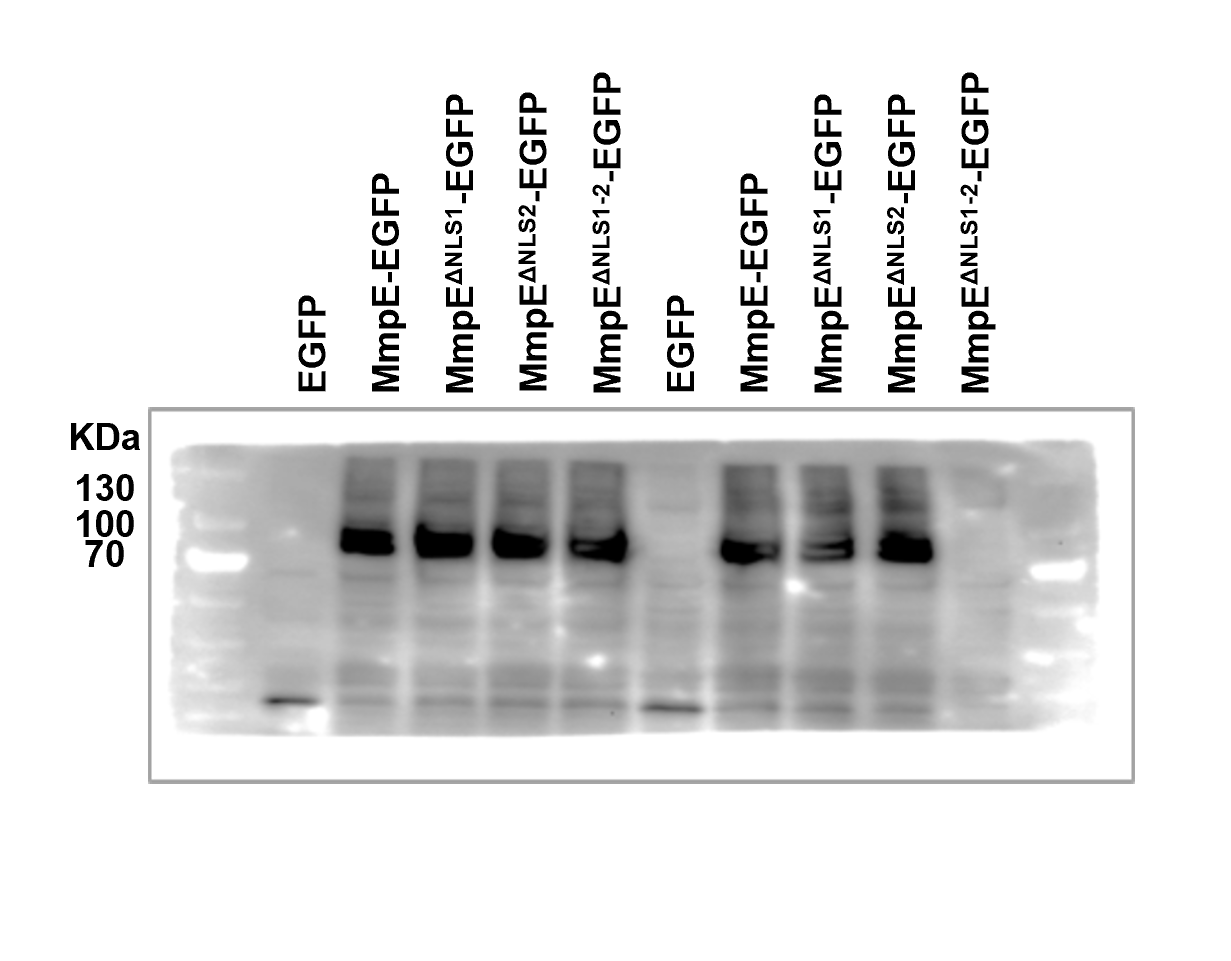

Supplement: Figure 1—source data 2. [file elife-108037-fig1-data2.zip › eLife-108037R1-Figure 1-sourse data and notes/Figure 1F-MmpE-GFP-3.tif]

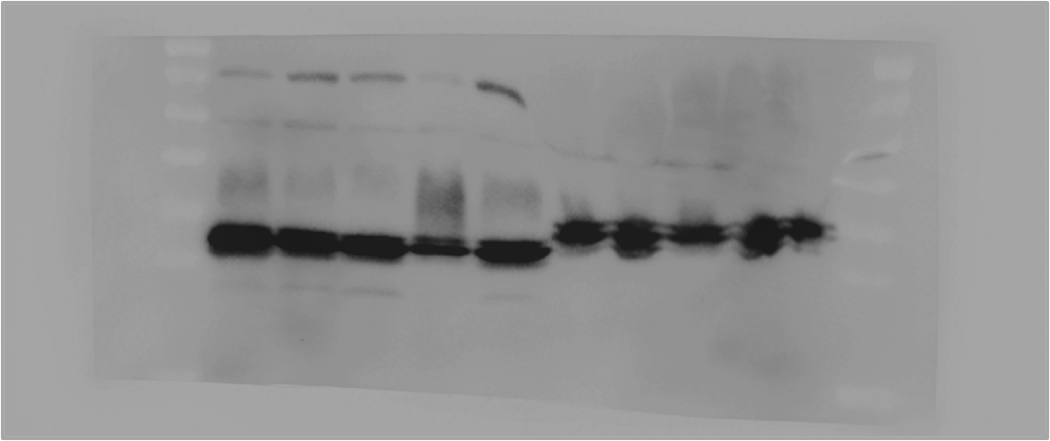

Supplement: Figure 1—figure supplement 1—source data 1. [file elife-108037-fig1-figsupp1-data1.zip › eLife-108037R1-Figure1-figure supplement 1/Figure1-figure supplement 1C-Ag85B.tif]

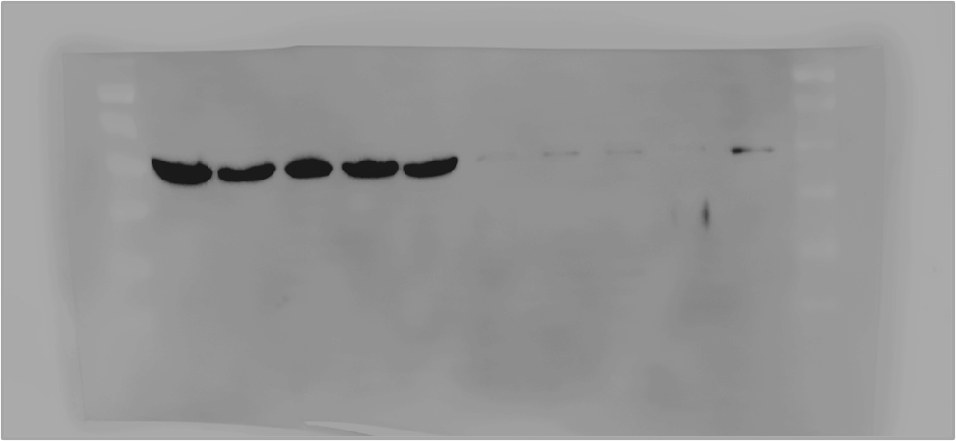

Supplement: Figure 1—figure supplement 1—source data 1. [file elife-108037-fig1-figsupp1-data1.zip › eLife-108037R1-Figure1-figure supplement 1/Figure1-figure supplement 1C-GlpX.tif]

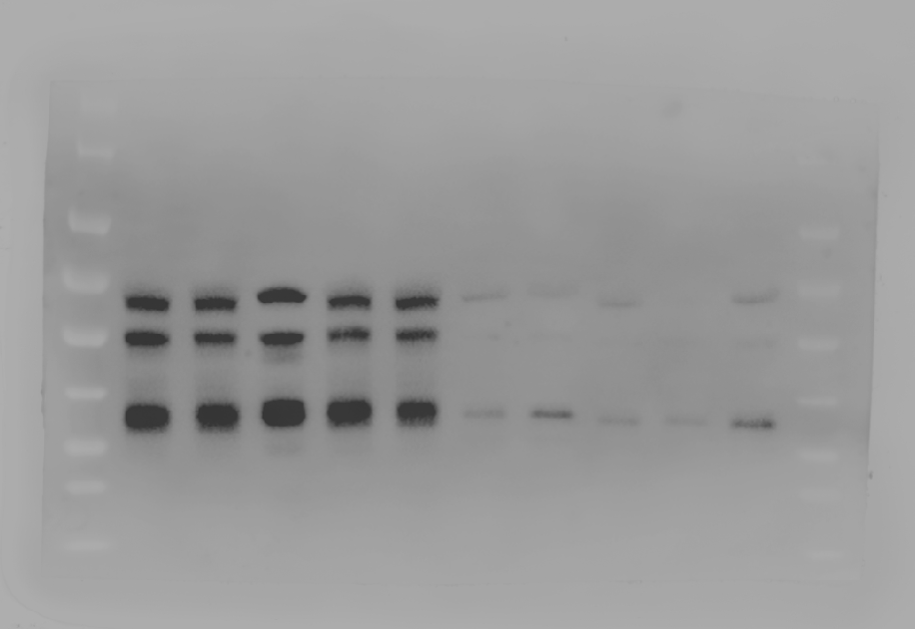

Supplement: Figure 1—figure supplement 1—source data 1. [file elife-108037-fig1-figsupp1-data1.zip › eLife-108037R1-Figure1-figure supplement 1/Figure1-figure supplement 1C-MmpE-Flag.tif]

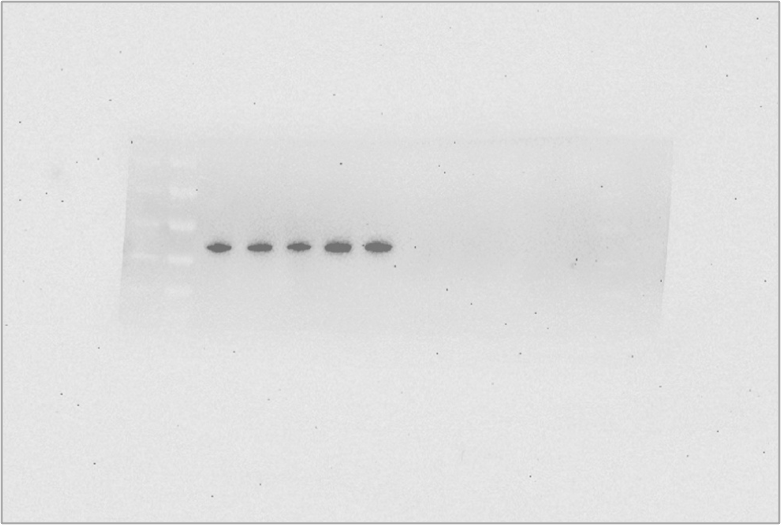

Supplement: Figure 1—figure supplement 1—source data 1. [file elife-108037-fig1-figsupp1-data1.zip › eLife-108037R1-Figure1-figure supplement 1/Figure1-figure supplement 1D-b-actin.tif]

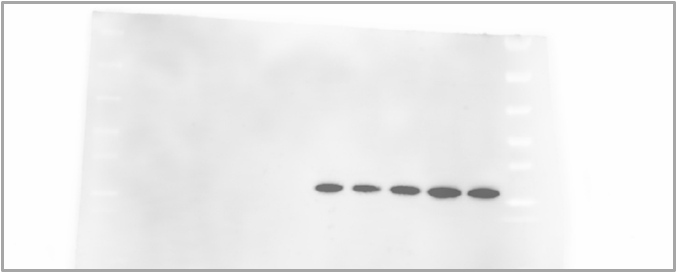

Supplement: Figure 1—figure supplement 1—source data 1. [file elife-108037-fig1-figsupp1-data1.zip › eLife-108037R1-Figure1-figure supplement 1/Figure1-figure supplement 1D-H3.tif]

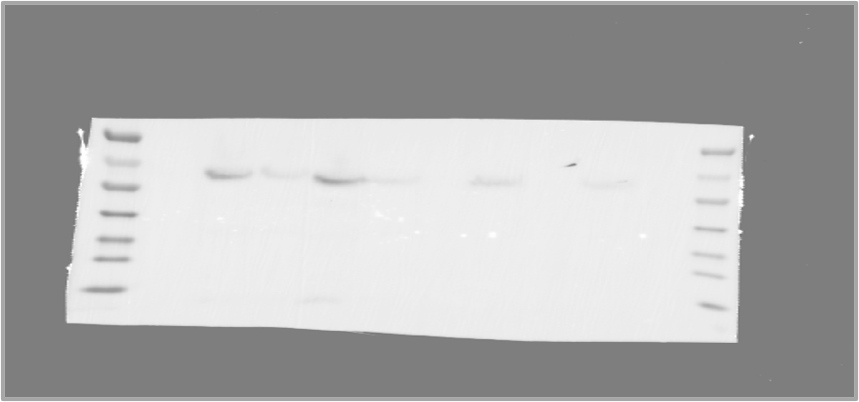

Supplement: Figure 1—figure supplement 1—source data 1. [file elife-108037-fig1-figsupp1-data1.zip › eLife-108037R1-Figure1-figure supplement 1/Figure1-figure supplement 1D-MmpE-Flag.tif]

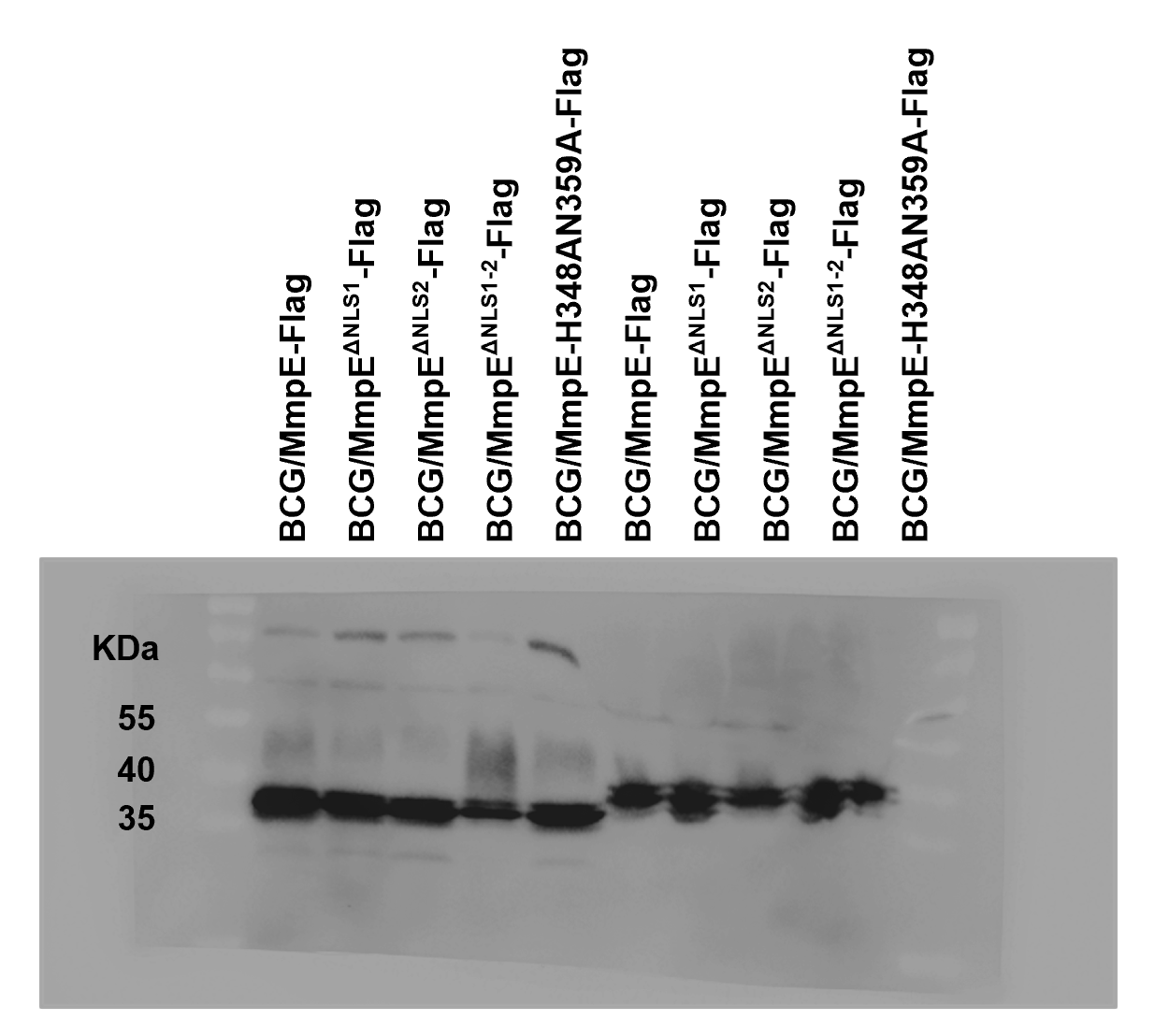

Supplement: Figure 1—figure supplement 1—source data 2. [file elife-108037-fig1-figsupp1-data2.zip › eLife-108037R1-Figure1-figure supplement 1 and notes/Figure1-figure supplement 1C-Ag85B.tif]

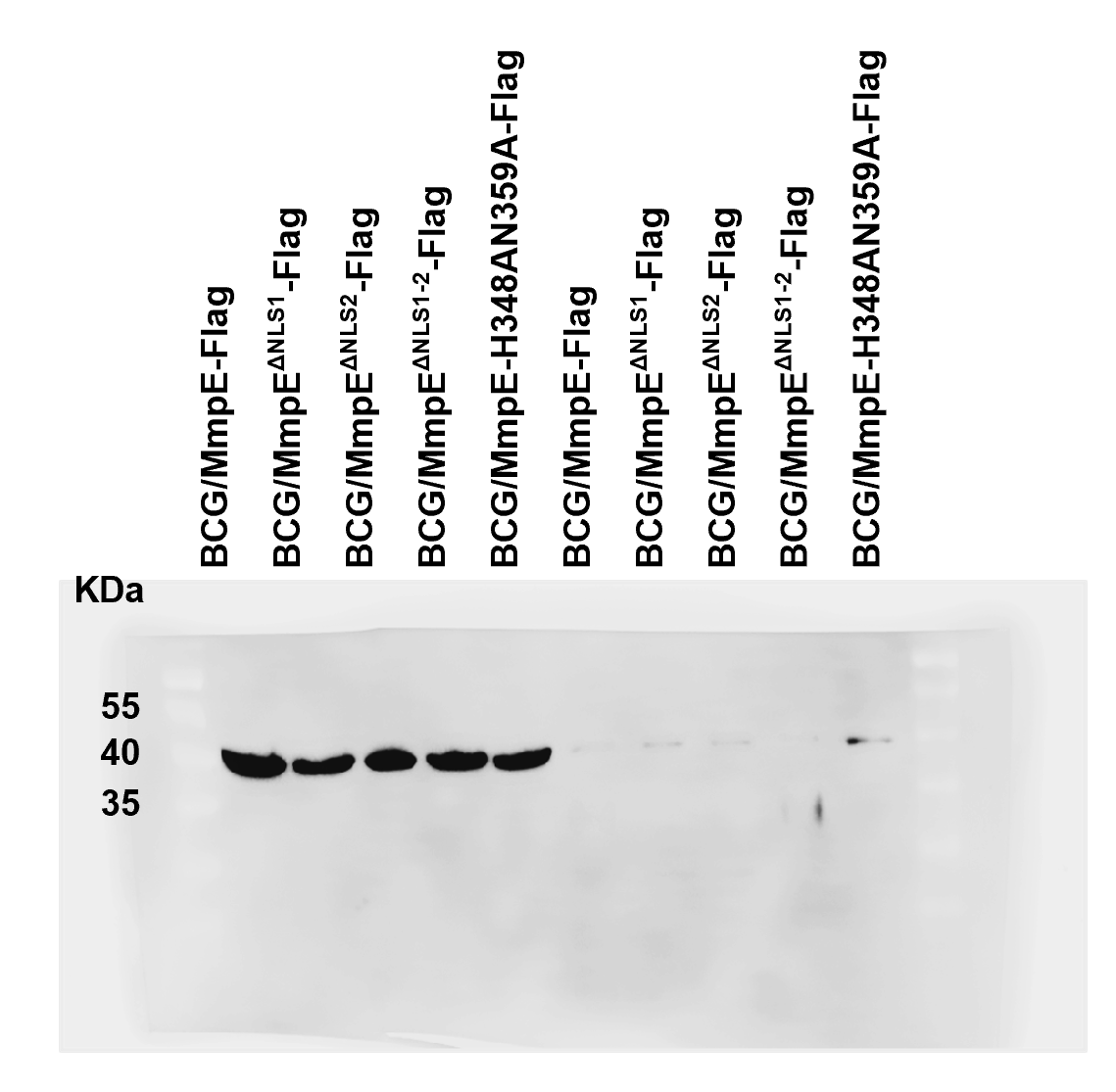

Supplement: Figure 1—figure supplement 1—source data 2. [file elife-108037-fig1-figsupp1-data2.zip › eLife-108037R1-Figure1-figure supplement 1 and notes/Figure1-figure supplement 1C-GlpX.tif]

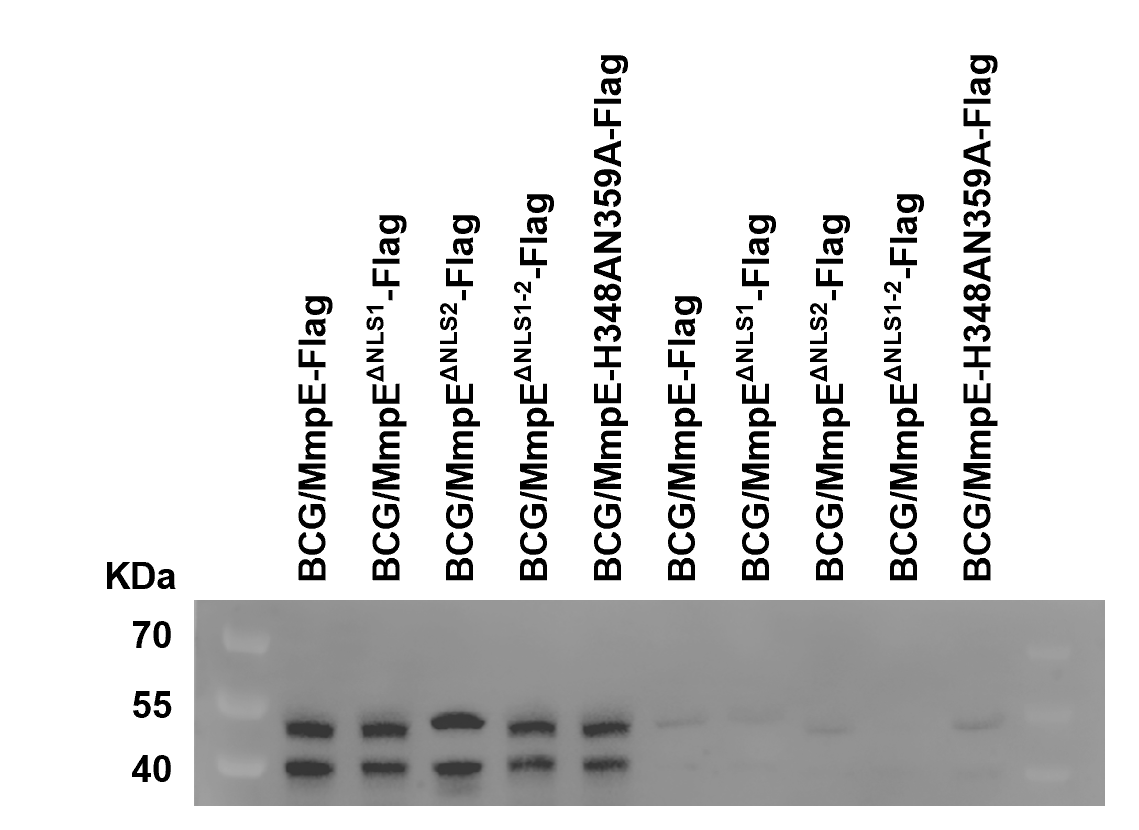

Supplement: Figure 1—figure supplement 1—source data 2. [file elife-108037-fig1-figsupp1-data2.zip › eLife-108037R1-Figure1-figure supplement 1 and notes/Figure1-figure supplement 1C-MmpE-Flag.tif]

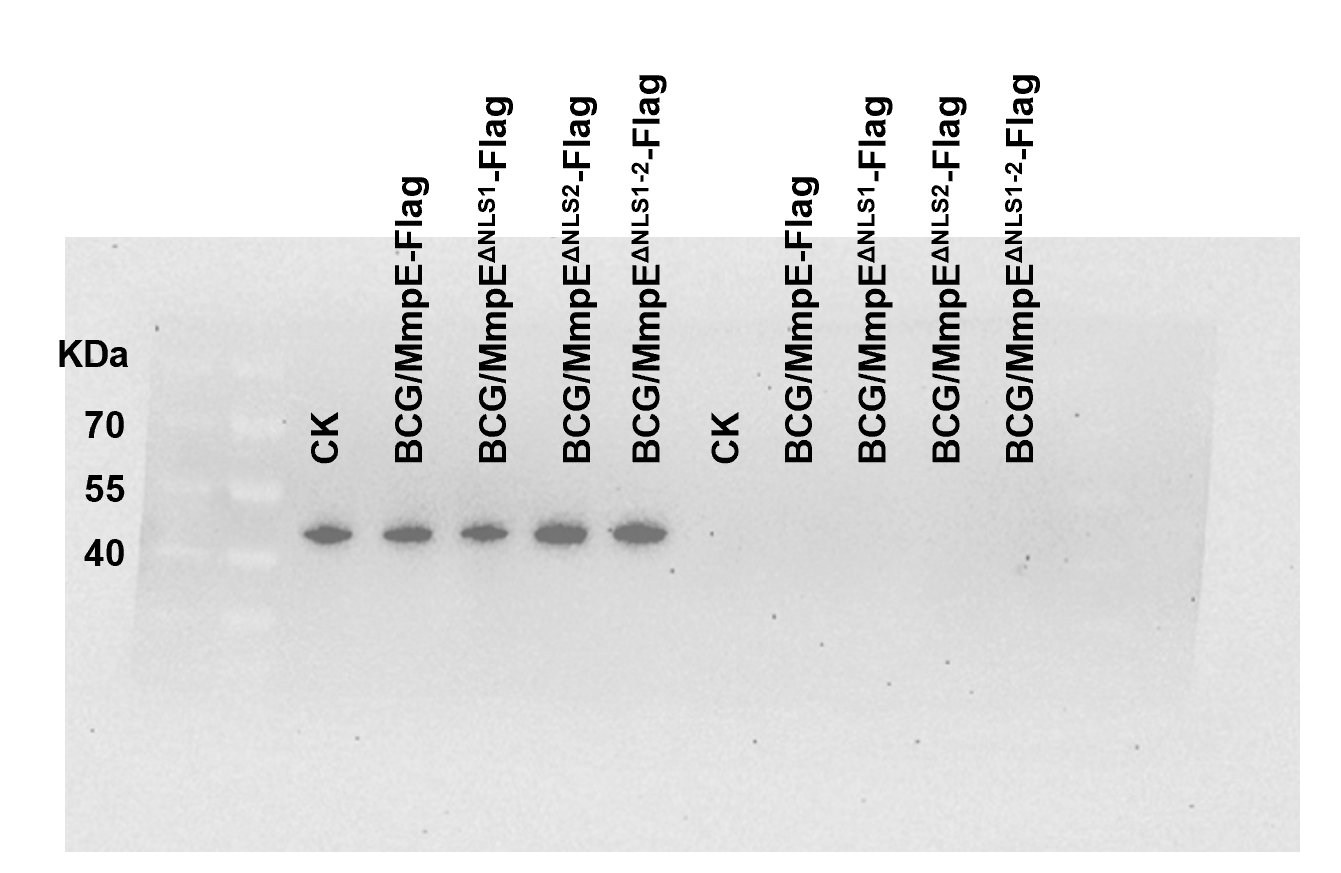

Supplement: Figure 1—figure supplement 1—source data 2. [file elife-108037-fig1-figsupp1-data2.zip › eLife-108037R1-Figure1-figure supplement 1 and notes/Figure1-figure supplement 1D-b-actin.tif]

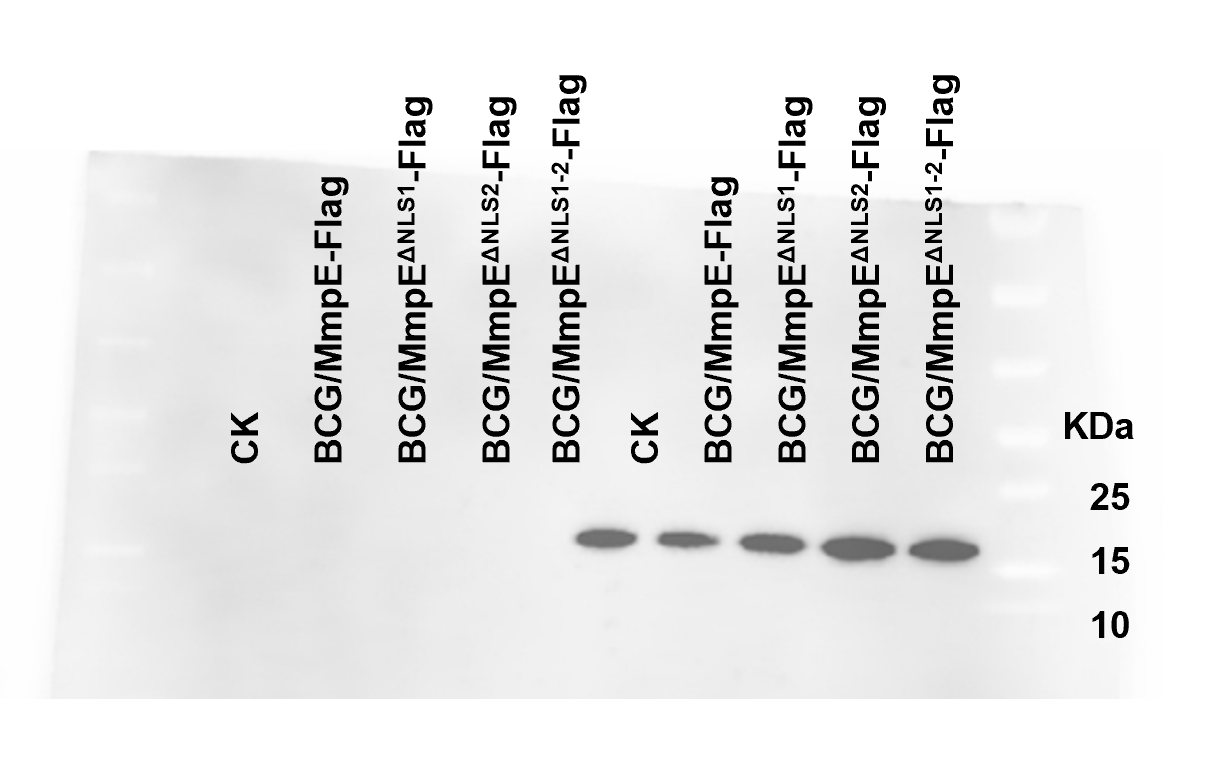

Supplement: Figure 1—figure supplement 1—source data 2. [file elife-108037-fig1-figsupp1-data2.zip › eLife-108037R1-Figure1-figure supplement 1 and notes/Figure1-figure supplement 1D-H3.tif]

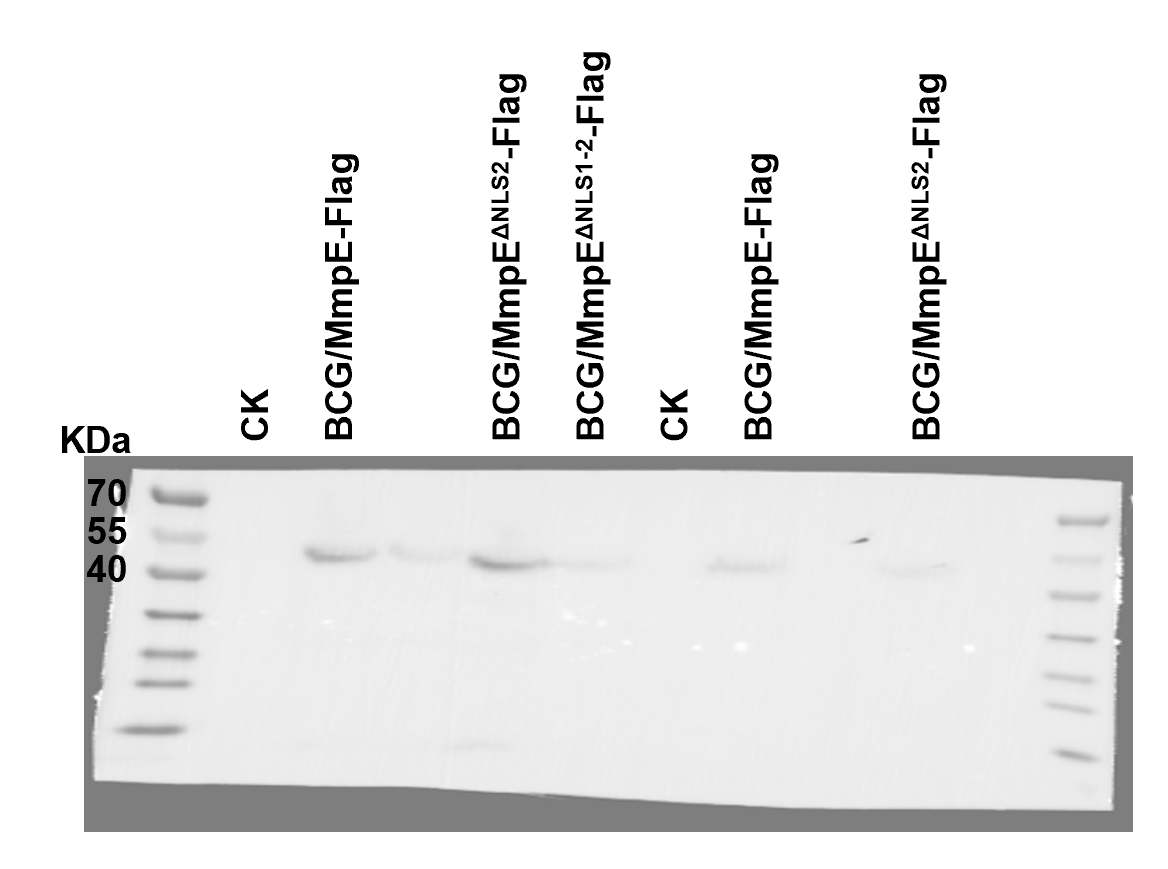

Supplement: Figure 1—figure supplement 1—source data 2. [file elife-108037-fig1-figsupp1-data2.zip › eLife-108037R1-Figure1-figure supplement 1 and notes/Figure1-figure supplement 1D-MmpE-Flag.tif]

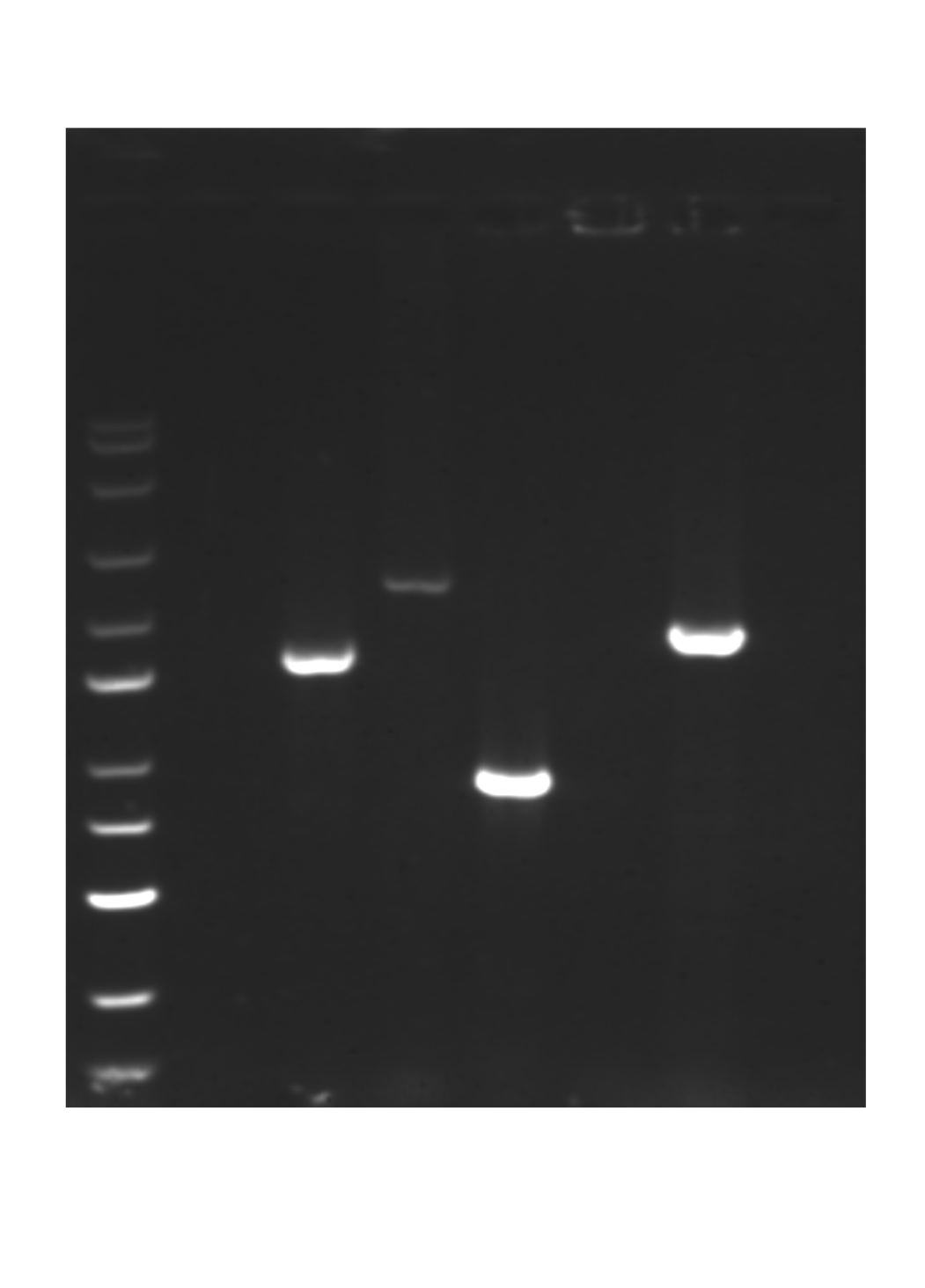

Supplement: Figure 3—figure supplement 1—source data 1. [file elife-108037-fig3-figsupp1-data1.zip › eLife-108037R1-Figure3-figure supplement 1/Figure3-figure supplement 1A-PCR.tif]

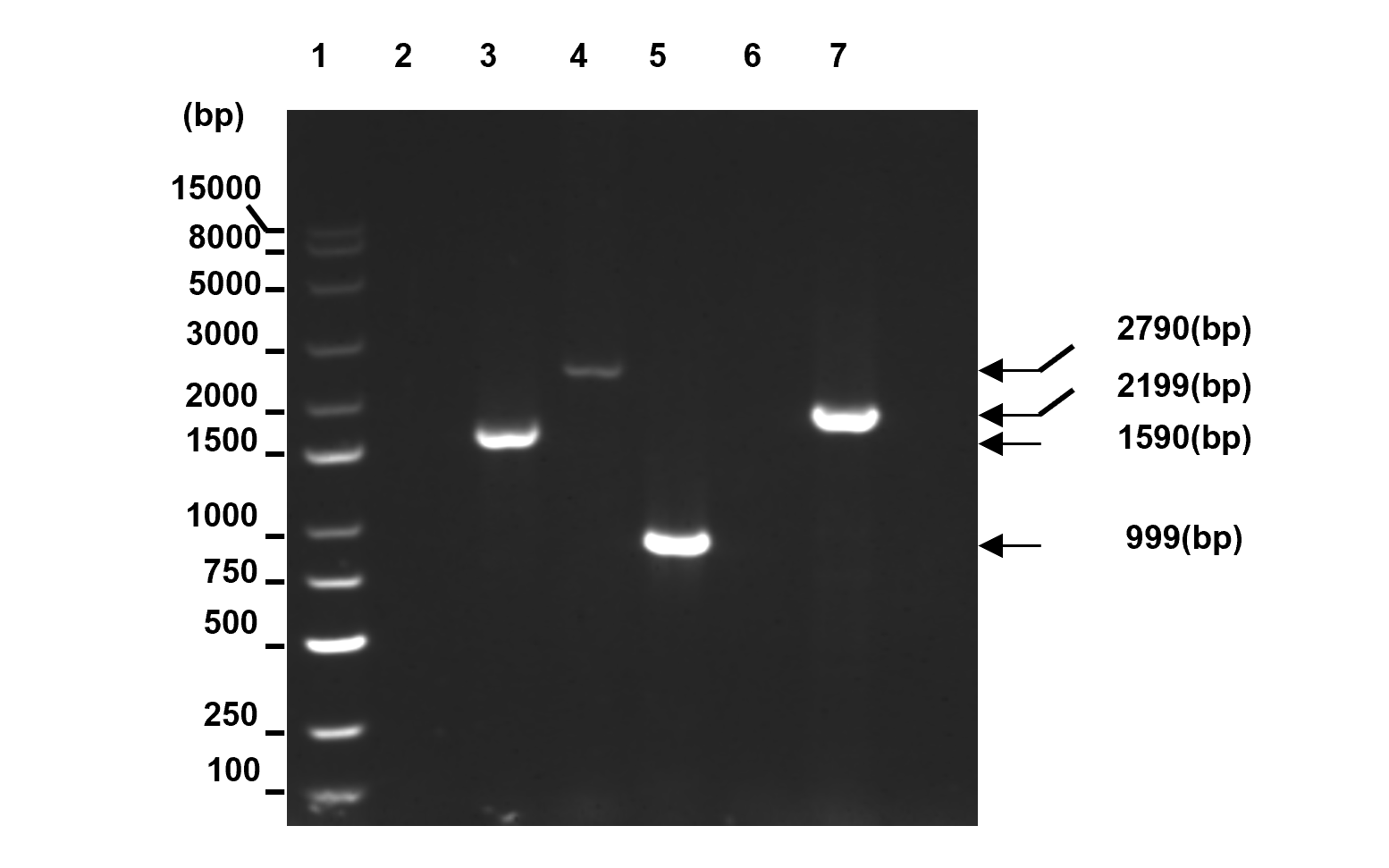

Supplement: Figure 3—figure supplement 1—source data 2. [file elife-108037-fig3-figsupp1-data2.zip › eLife-108037R1-Figure3-figure supplement 1 and notes/Figure3-figure supplement 1A-PCR.tif]

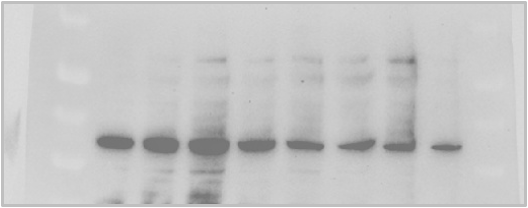

Supplement: Figure 5—source data 1. [file elife-108037-fig5-data1.zip › eLife-108037R1-Figure 5-sourse data/Figure 5E-AKT.tif]

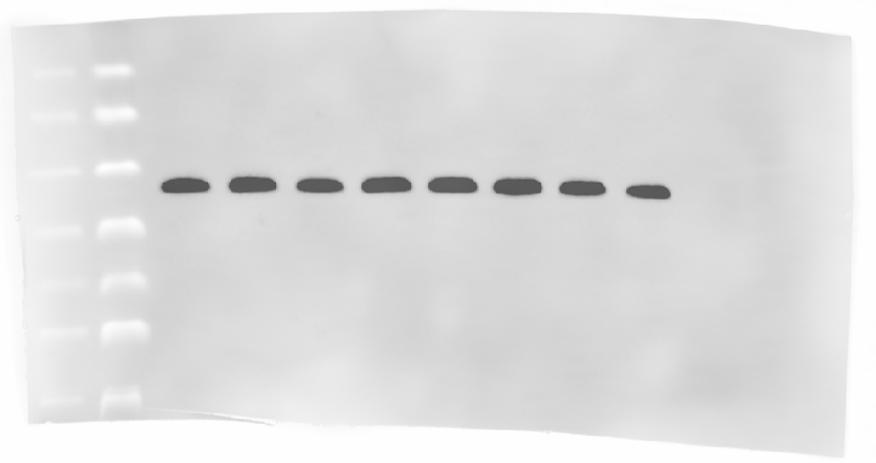

Supplement: Figure 5—source data 1. [file elife-108037-fig5-data1.zip › eLife-108037R1-Figure 5-sourse data/Figure 5E-b-actin.tif]

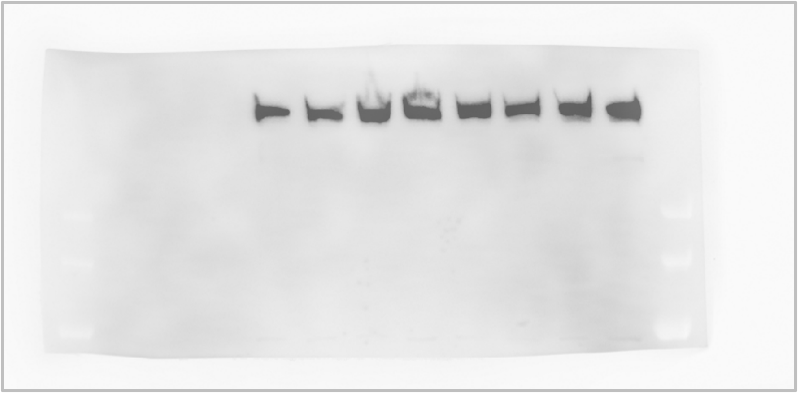

Supplement: Figure 5—source data 1. [file elife-108037-fig5-data1.zip › eLife-108037R1-Figure 5-sourse data/Figure 5E-mTOR.tif]

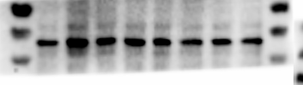

Supplement: Figure 5—source data 1. [file elife-108037-fig5-data1.zip › eLife-108037R1-Figure 5-sourse data/Figure 5E-p-AKT-1.tif]

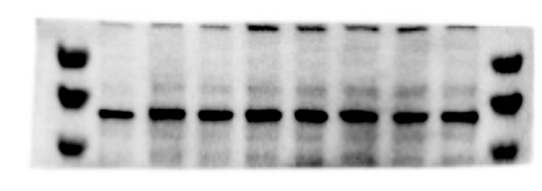

Supplement: Figure 5—source data 1. [file elife-108037-fig5-data1.zip › eLife-108037R1-Figure 5-sourse data/Figure 5E-p-AKT-2.tif]

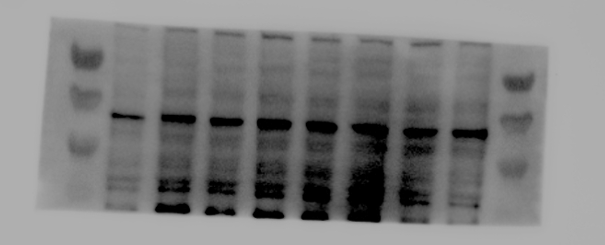

Supplement: Figure 5—source data 1. [file elife-108037-fig5-data1.zip › eLife-108037R1-Figure 5-sourse data/Figure 5E-p-AKT-3.tif]

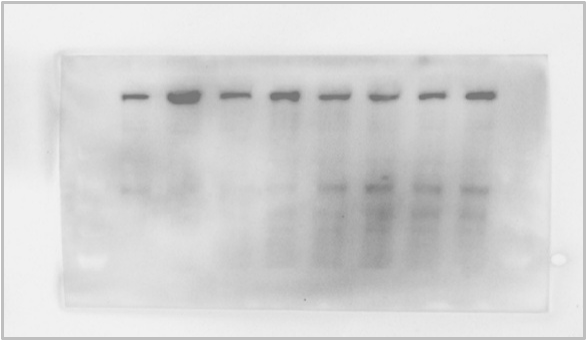

Supplement: Figure 5—source data 1. [file elife-108037-fig5-data1.zip › eLife-108037R1-Figure 5-sourse data/Figure 5E-p-mTOR(S2448)-1.tif]

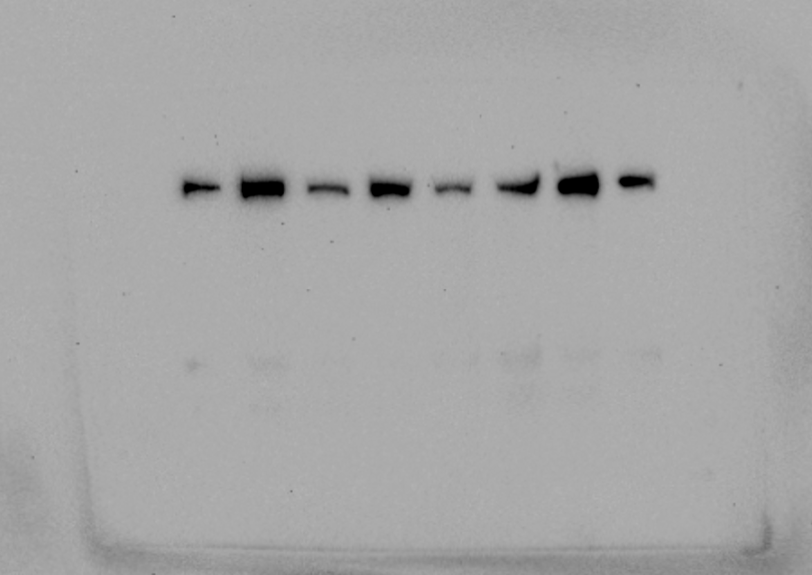

Supplement: Figure 5—source data 1. [file elife-108037-fig5-data1.zip › eLife-108037R1-Figure 5-sourse data/Figure 5E-p-mTOR(S2448)-2.tif]

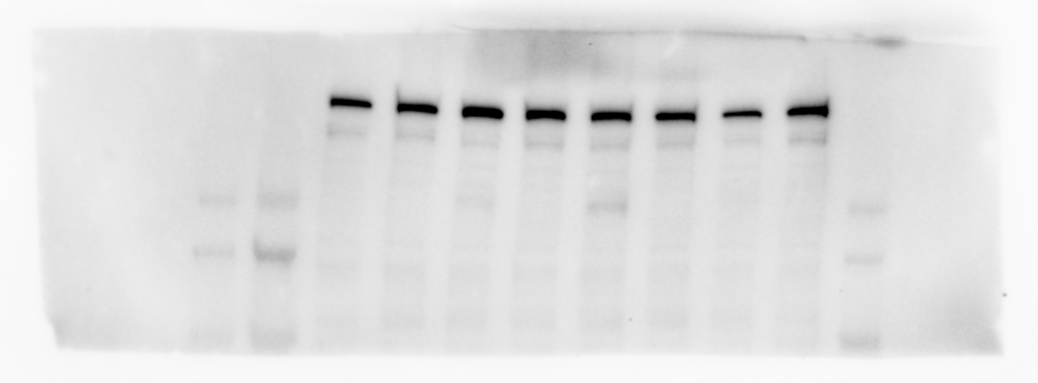

Supplement: Figure 5—source data 1. [file elife-108037-fig5-data1.zip › eLife-108037R1-Figure 5-sourse data/Figure 5E-p-mTOR(S2448)-3.tif]

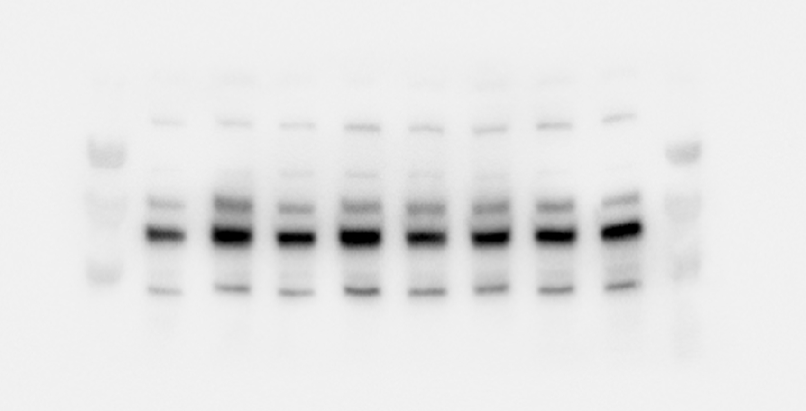

Supplement: Figure 5—source data 1. [file elife-108037-fig5-data1.zip › eLife-108037R1-Figure 5-sourse data/Figure 5E-p-p70(S6K)-1.tif]

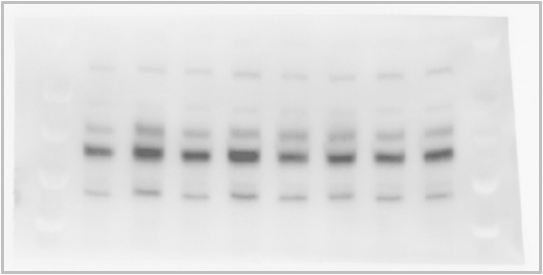

Supplement: Figure 5—source data 1. [file elife-108037-fig5-data1.zip › eLife-108037R1-Figure 5-sourse data/Figure 5E-p-p70(S6K)-2.tif]

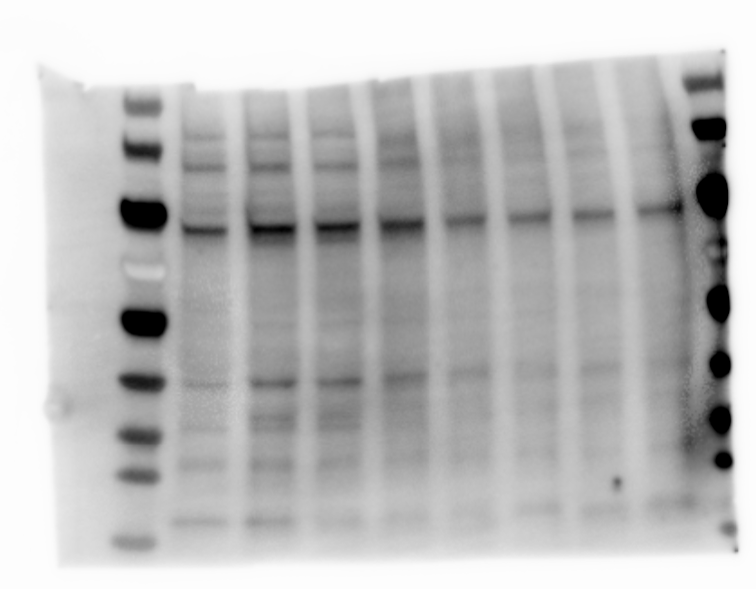

Supplement: Figure 5—source data 1. [file elife-108037-fig5-data1.zip › eLife-108037R1-Figure 5-sourse data/Figure 5E-p-p70(S6K)-3.tif]

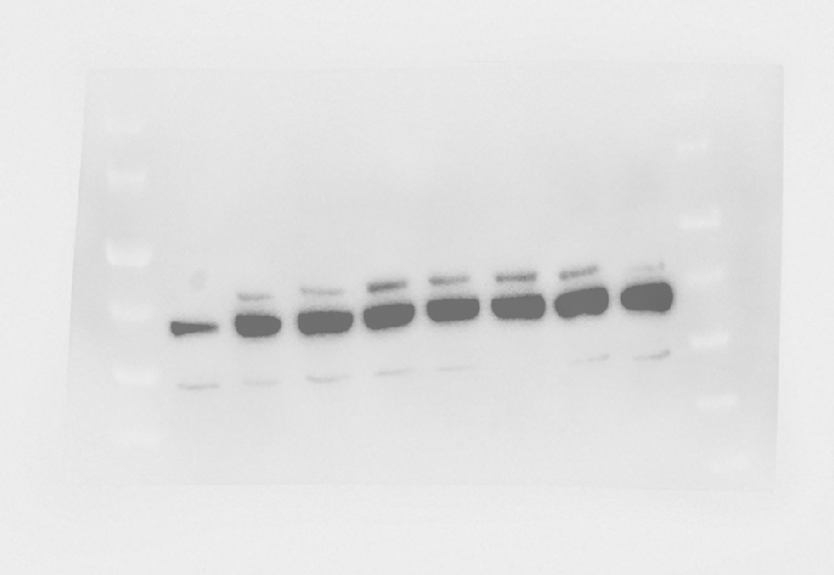

Supplement: Figure 5—source data 1. [file elife-108037-fig5-data1.zip › eLife-108037R1-Figure 5-sourse data/Figure 5E-p70(S6K).tif]

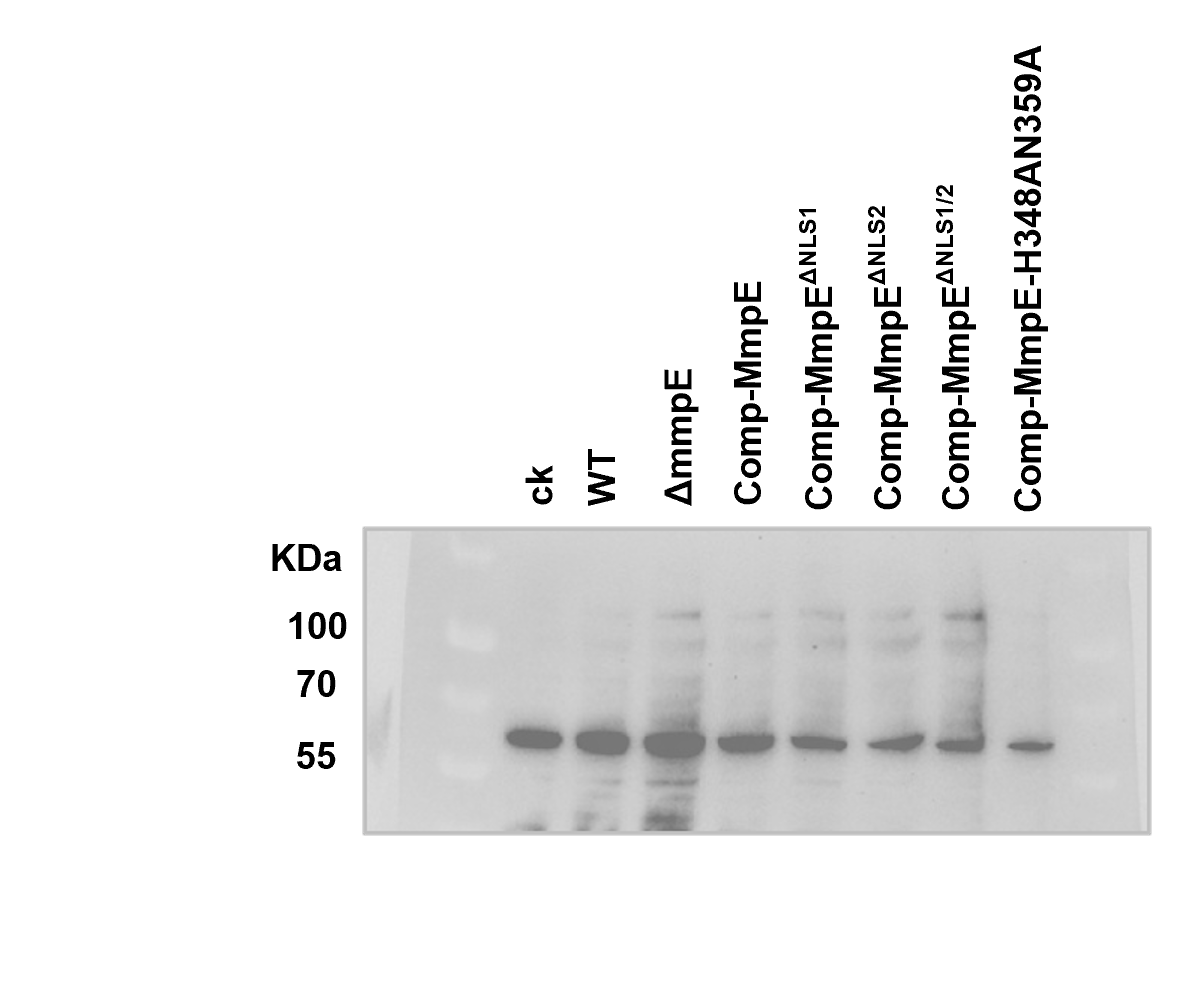

Supplement: Figure 5—source data 2. [file elife-108037-fig5-data2.zip › eLife-108037R1-Figure 5-sourse data and notes/Figure 5E-AKT.tif]

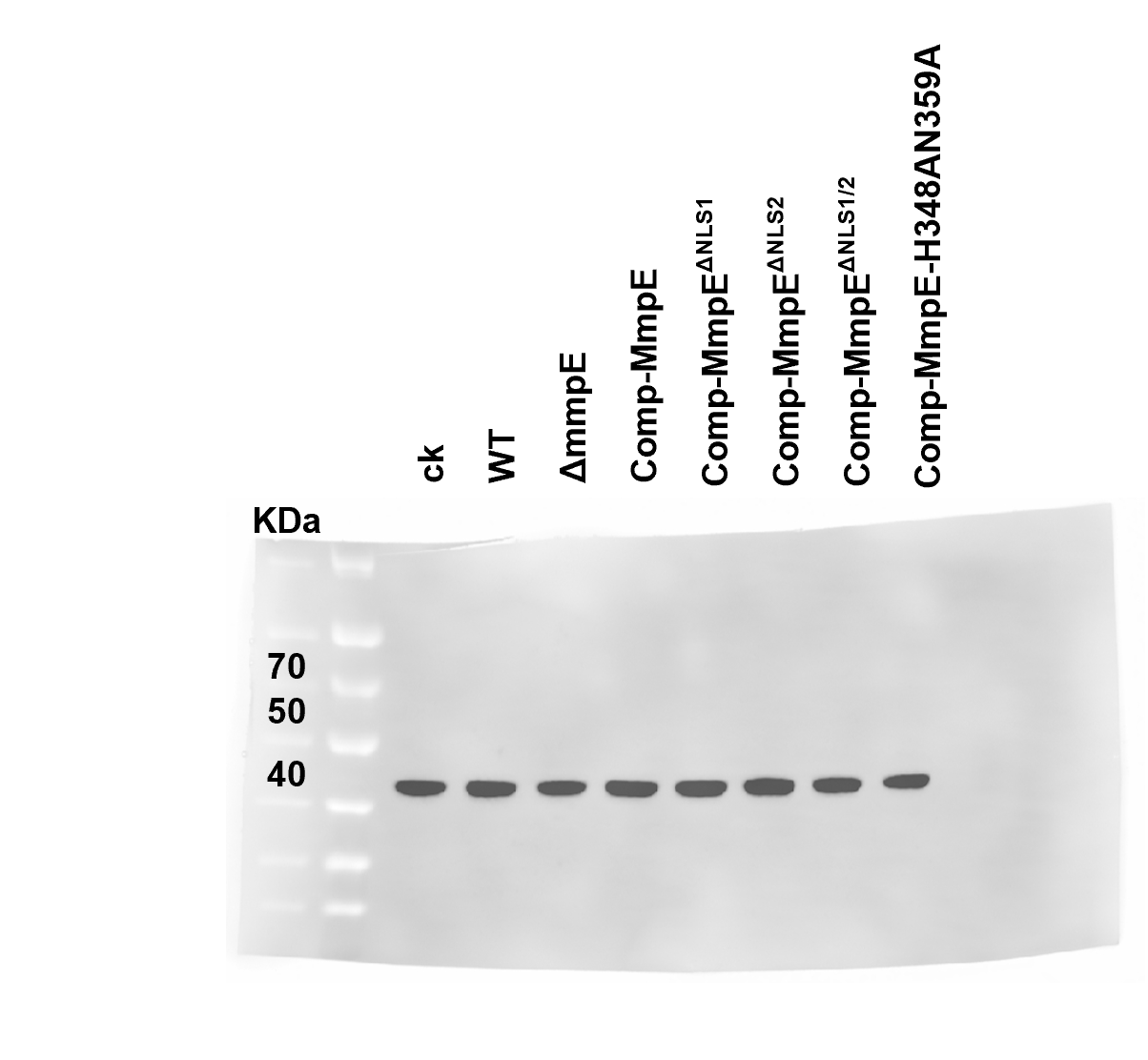

Supplement: Figure 5—source data 2. [file elife-108037-fig5-data2.zip › eLife-108037R1-Figure 5-sourse data and notes/Figure 5E-b-actin.tif]

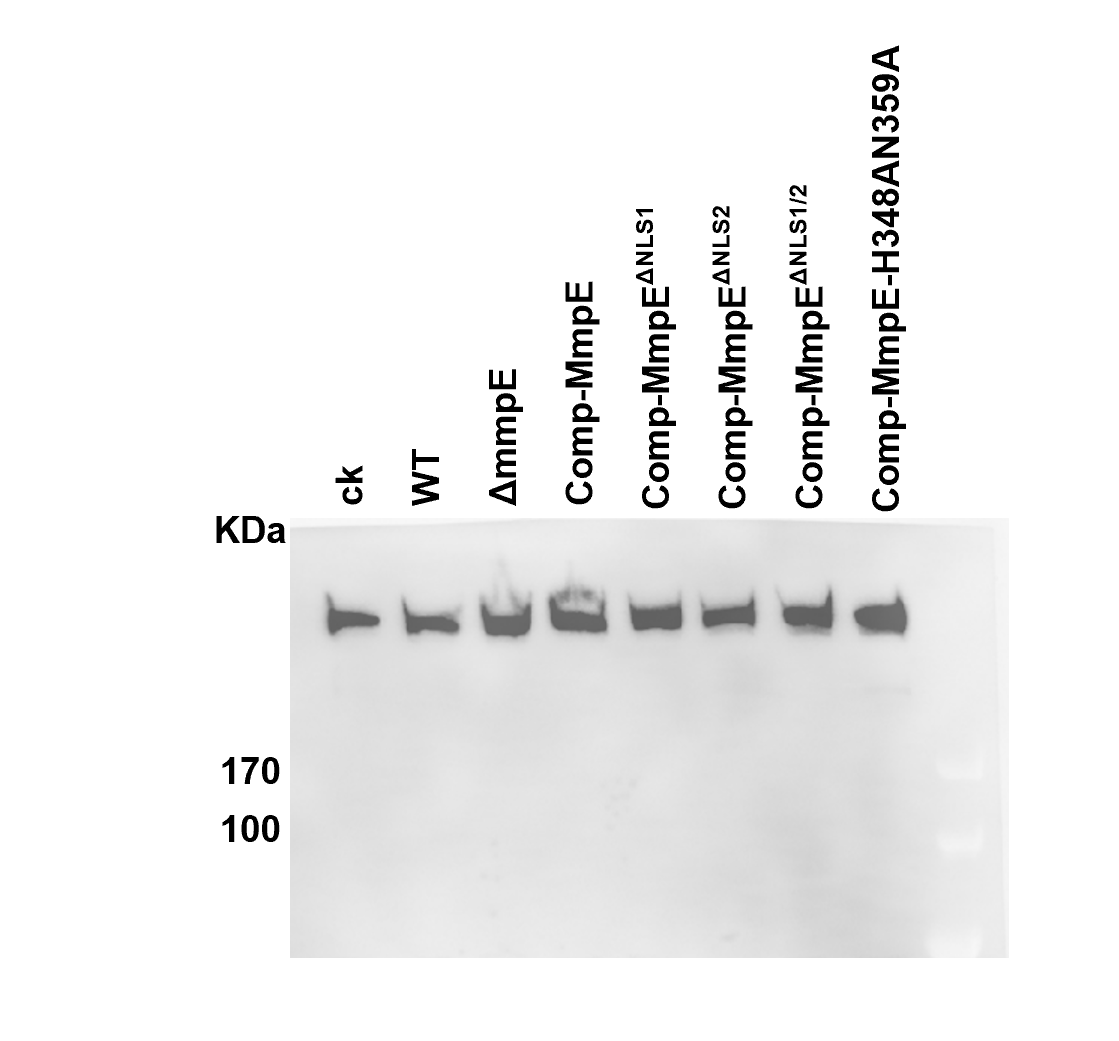

Supplement: Figure 5—source data 2. [file elife-108037-fig5-data2.zip › eLife-108037R1-Figure 5-sourse data and notes/Figure 5E-mTOR.tif]

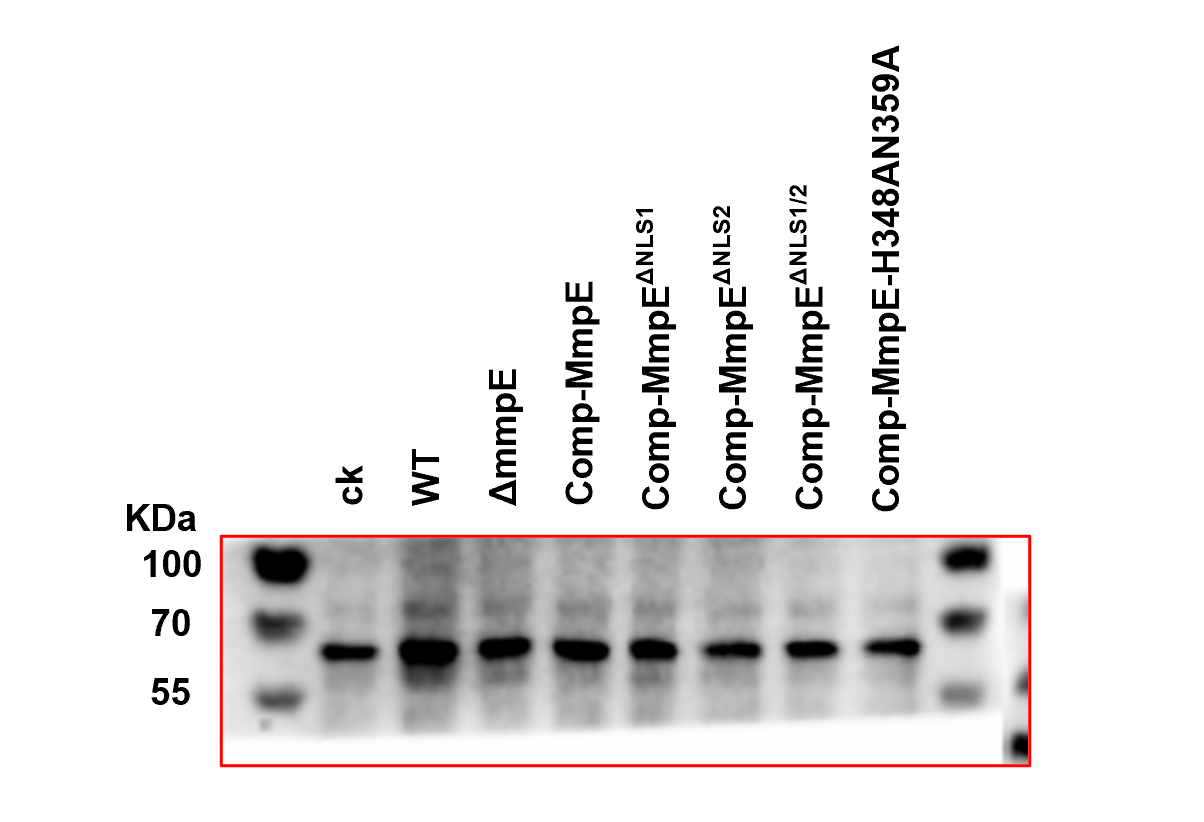

Supplement: Figure 5—source data 2. [file elife-108037-fig5-data2.zip › eLife-108037R1-Figure 5-sourse data and notes/Figure 5E-p-AKT-1.tif]

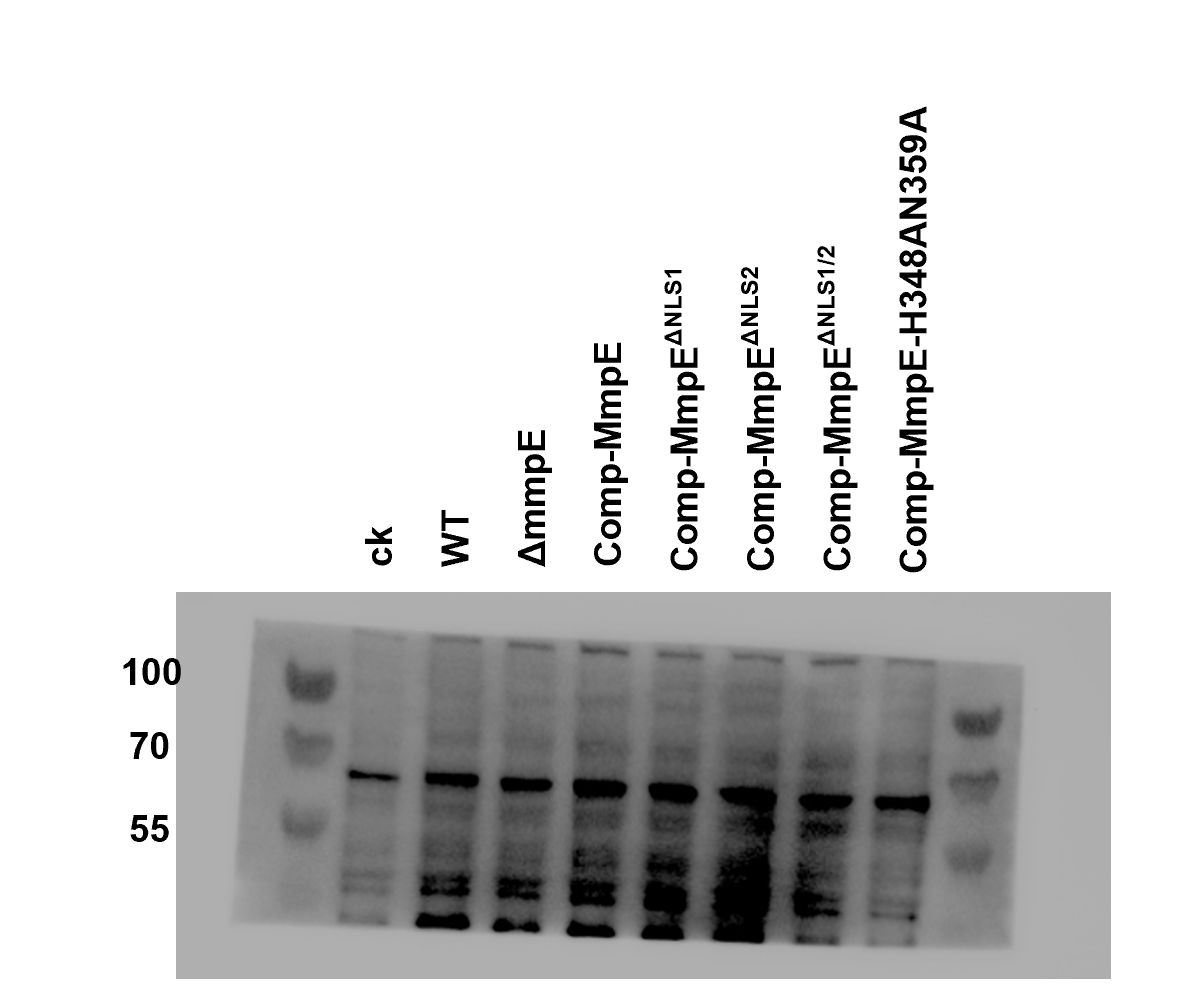

Supplement: Figure 5—source data 2. [file elife-108037-fig5-data2.zip › eLife-108037R1-Figure 5-sourse data and notes/Figure 5E-p-AKT-2.tif]

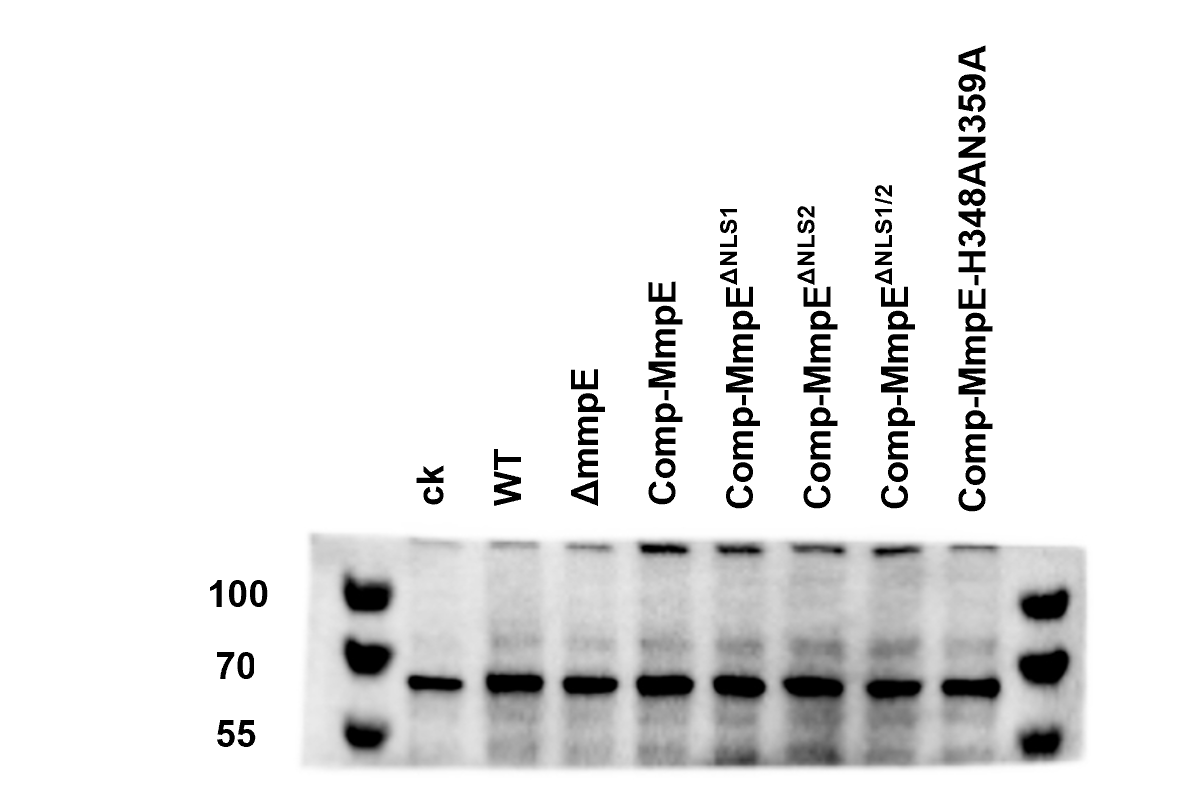

Supplement: Figure 5—source data 2. [file elife-108037-fig5-data2.zip › eLife-108037R1-Figure 5-sourse data and notes/Figure 5E-p-AKT-3.tif]

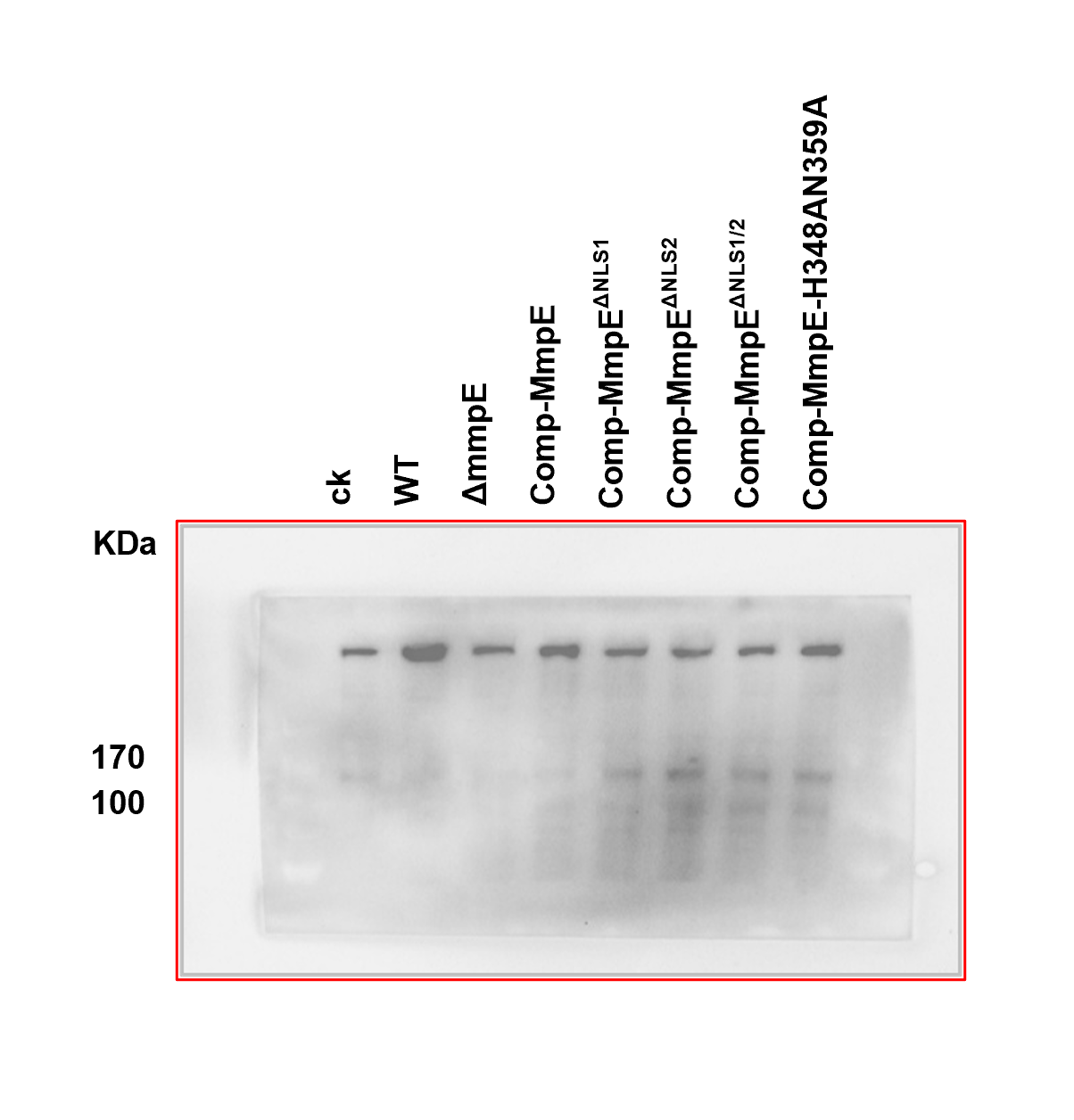

Supplement: Figure 5—source data 2. [file elife-108037-fig5-data2.zip › eLife-108037R1-Figure 5-sourse data and notes/Figure 5E-p-mTOR(S2448)-1.tif]

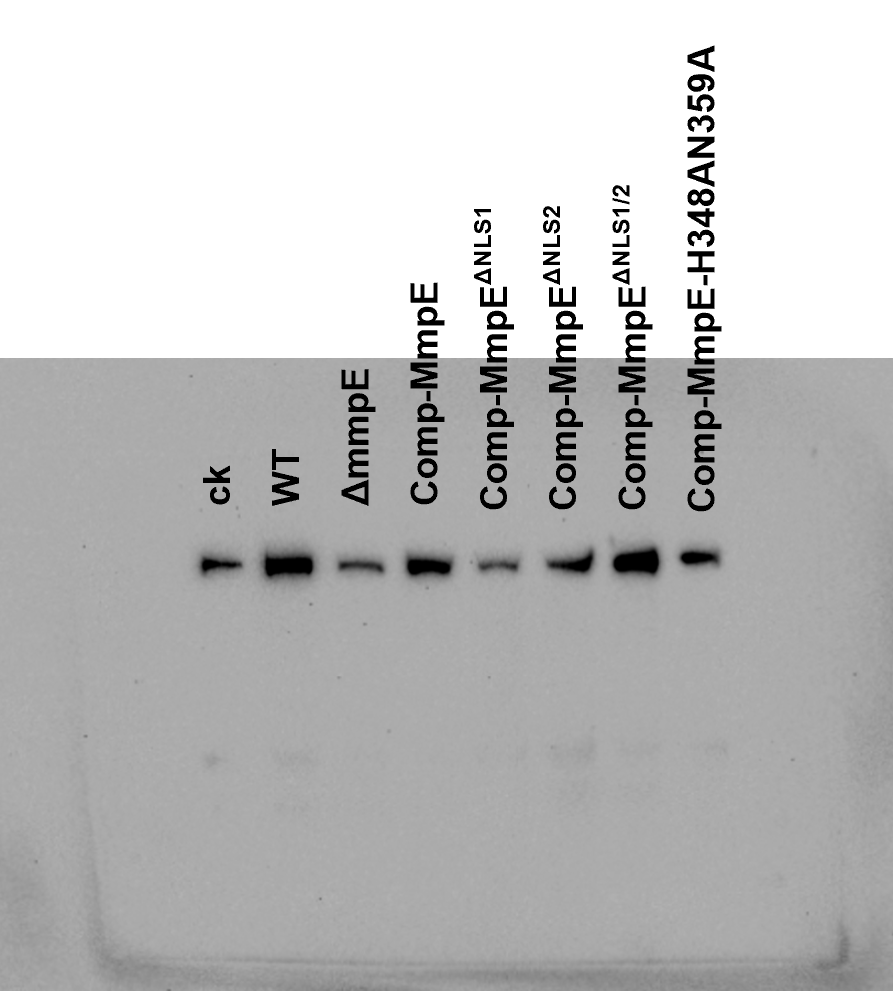

Supplement: Figure 5—source data 2. [file elife-108037-fig5-data2.zip › eLife-108037R1-Figure 5-sourse data and notes/Figure 5E-p-mTOR(S2448)-2.tif]

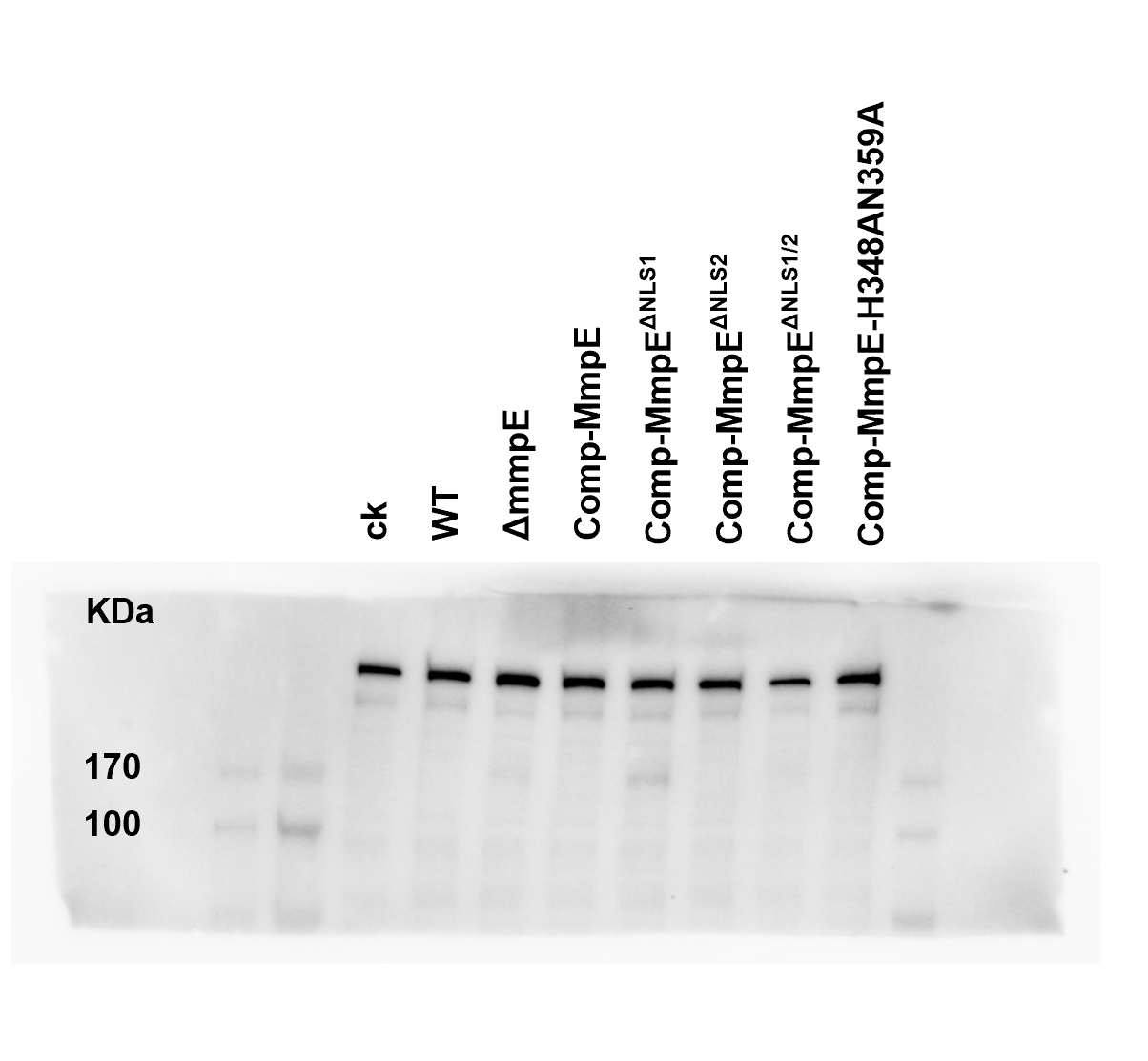

Supplement: Figure 5—source data 2. [file elife-108037-fig5-data2.zip › eLife-108037R1-Figure 5-sourse data and notes/Figure 5E-p-mTOR(S2448)-3.tif]

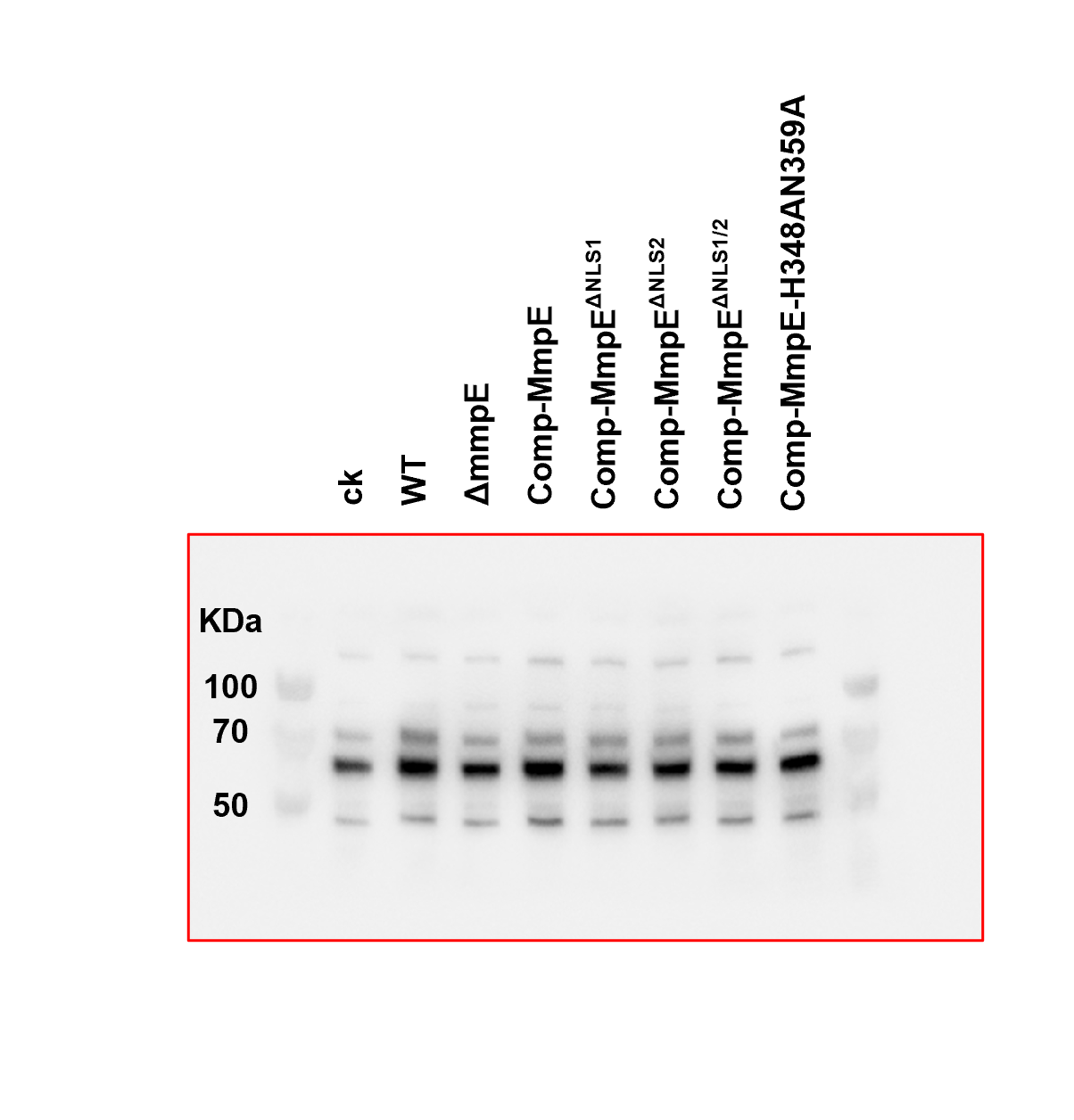

Supplement: Figure 5—source data 2. [file elife-108037-fig5-data2.zip › eLife-108037R1-Figure 5-sourse data and notes/Figure 5E-p-p70(S6K)-1.tif]

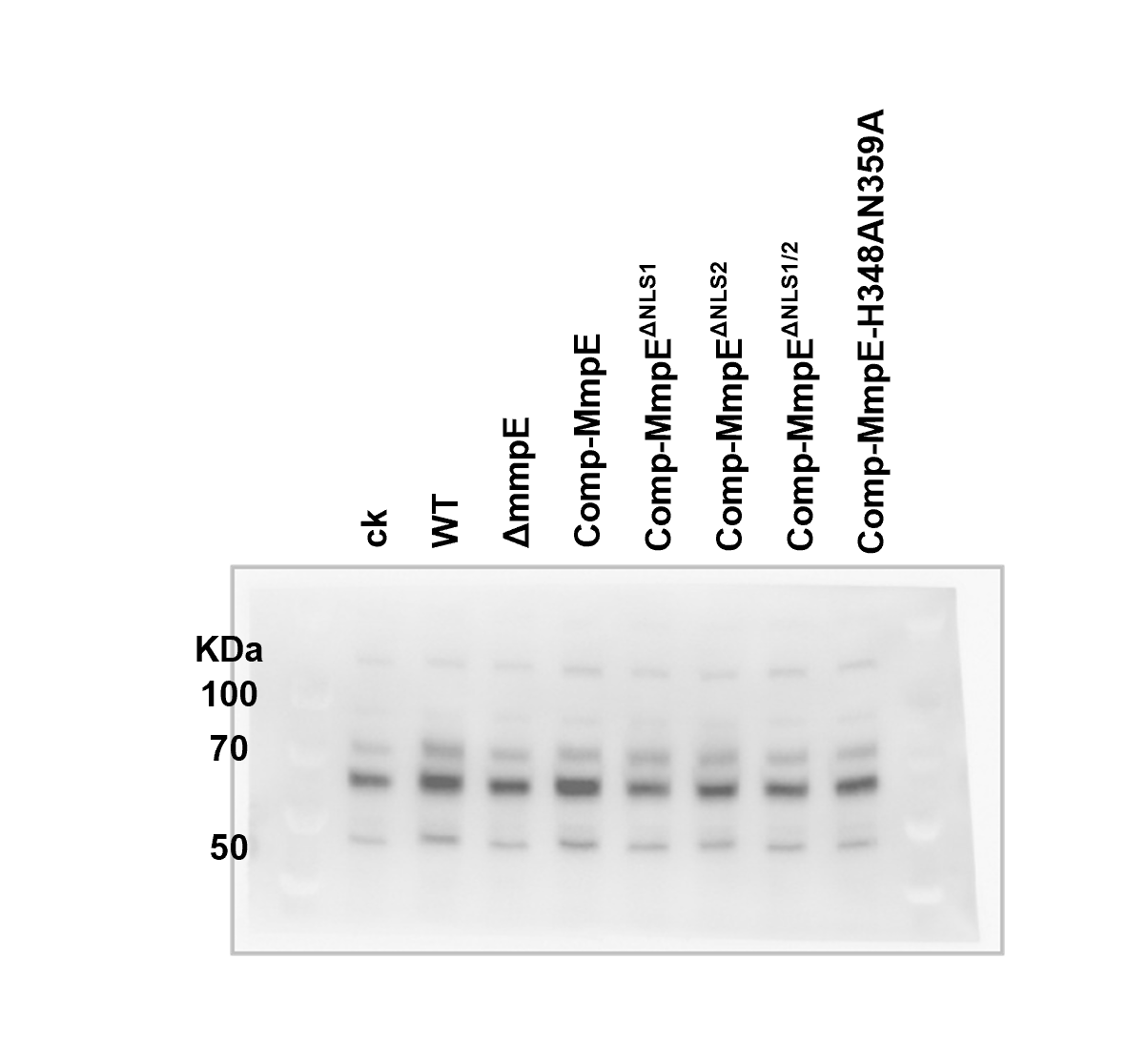

Supplement: Figure 5—source data 2. [file elife-108037-fig5-data2.zip › eLife-108037R1-Figure 5-sourse data and notes/Figure 5E-p-p70(S6K)-2.tif]

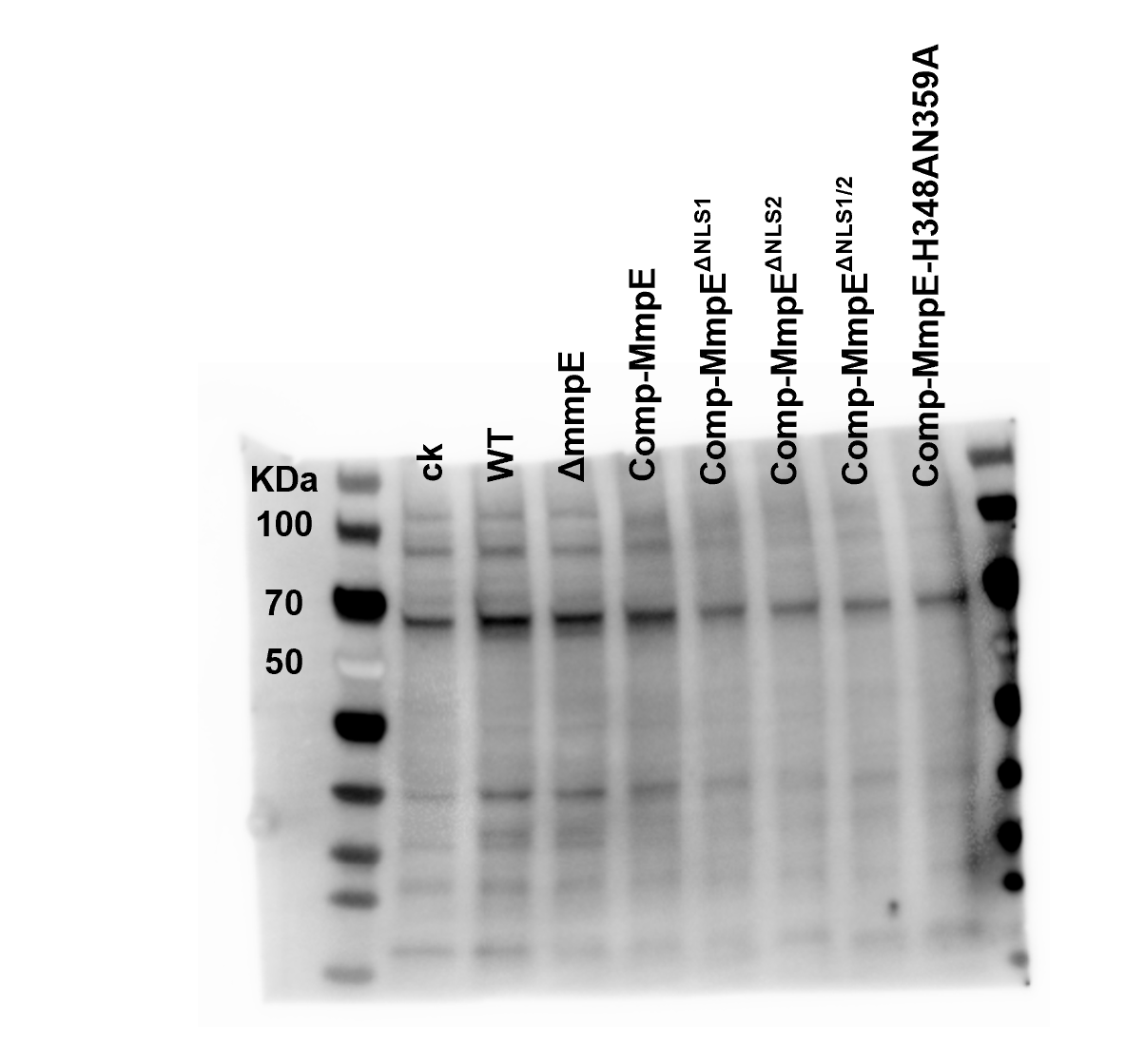

Supplement: Figure 5—source data 2. [file elife-108037-fig5-data2.zip › eLife-108037R1-Figure 5-sourse data and notes/Figure 5E-p-p70(S6K)-3.tif]

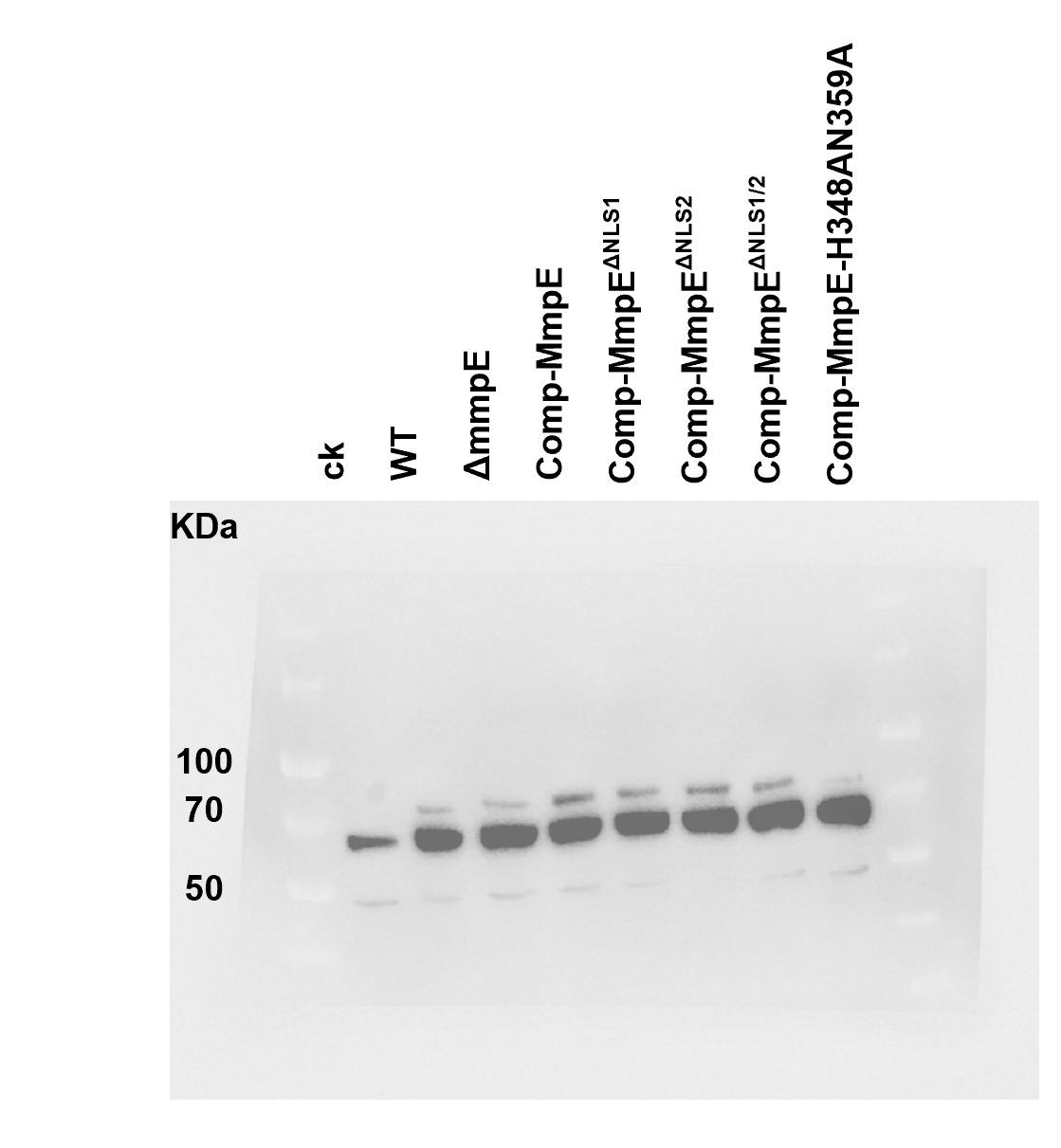

Supplement: Figure 5—source data 2. [file elife-108037-fig5-data2.zip › eLife-108037R1-Figure 5-sourse data and notes/Figure 5E-p70(S6K).tif]

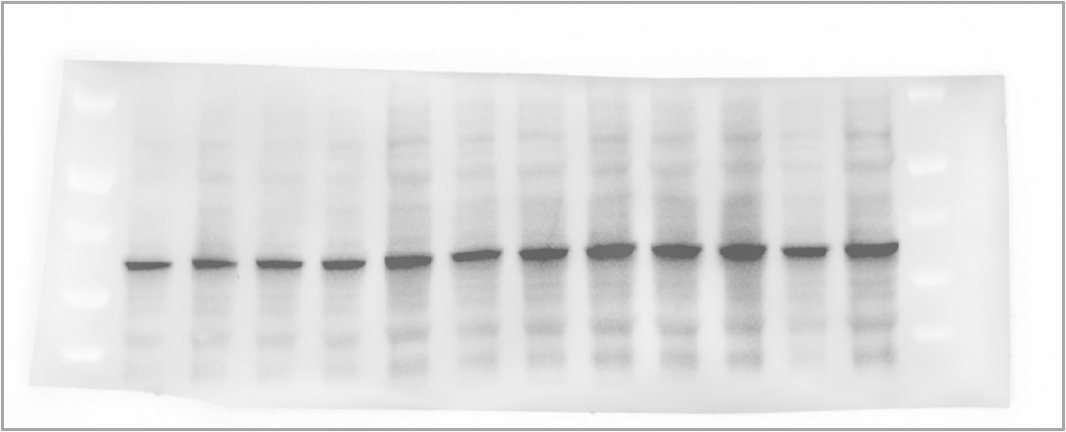

Supplement: Figure 5—figure supplement 1—source data 1. [file elife-108037-fig5-figsupp1-data1.zip › eLife-108037R1-Figure5-figure supplement 1/Figure5-figure supplement 1C-AKT.tif]

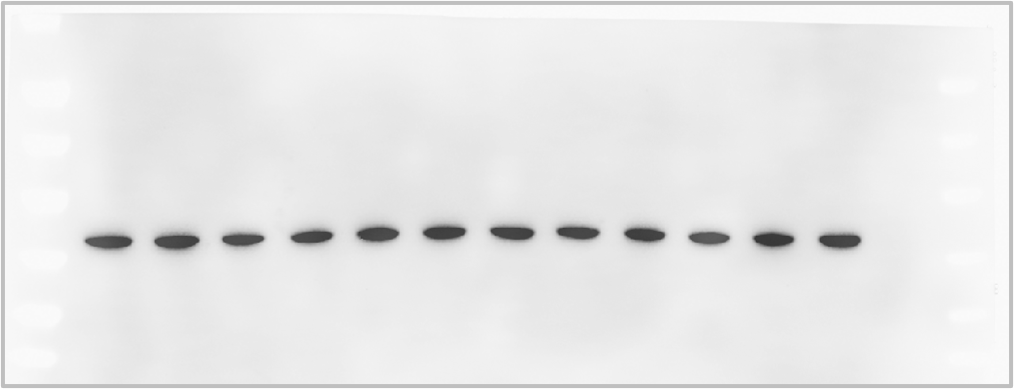

Supplement: Figure 5—figure supplement 1—source data 1. [file elife-108037-fig5-figsupp1-data1.zip › eLife-108037R1-Figure5-figure supplement 1/Figure5-figure supplement 1C-b-actin.tif]

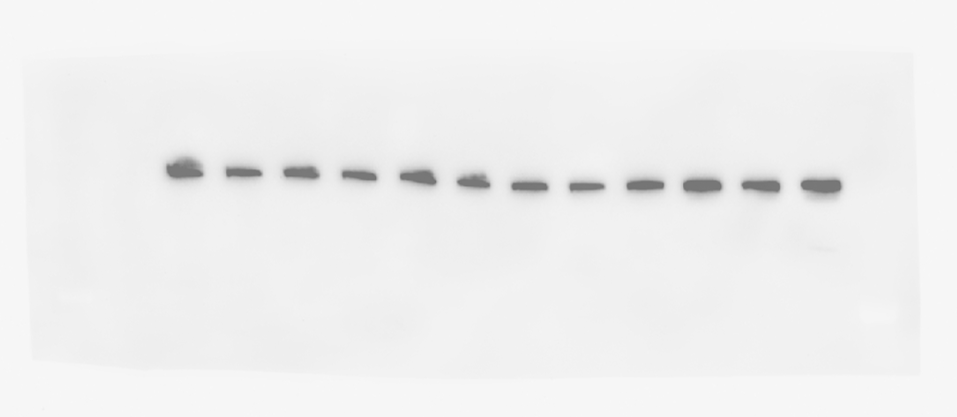

Supplement: Figure 5—figure supplement 1—source data 1. [file elife-108037-fig5-figsupp1-data1.zip › eLife-108037R1-Figure5-figure supplement 1/Figure5-figure supplement 1C-mTOR.tif]

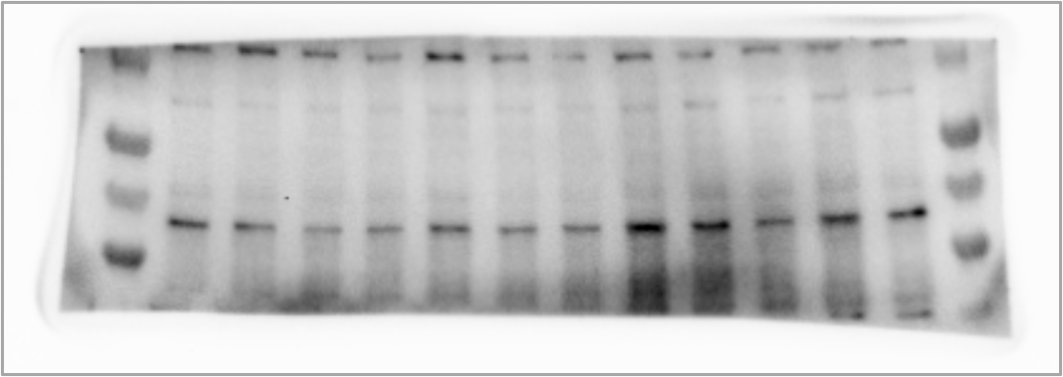

Supplement: Figure 5—figure supplement 1—source data 1. [file elife-108037-fig5-figsupp1-data1.zip › eLife-108037R1-Figure5-figure supplement 1/Figure5-figure supplement 1C-p-AKT-1.tif]

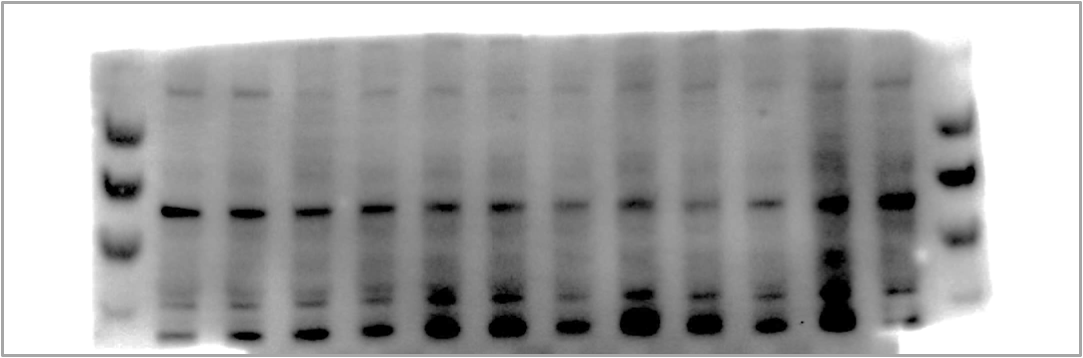

Supplement: Figure 5—figure supplement 1—source data 1. [file elife-108037-fig5-figsupp1-data1.zip › eLife-108037R1-Figure5-figure supplement 1/Figure5-figure supplement 1C-p-AKT-2.tif]

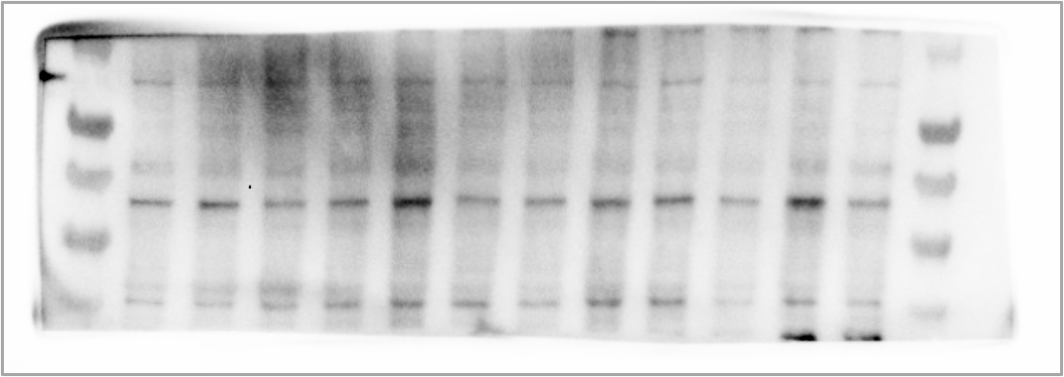

Supplement: Figure 5—figure supplement 1—source data 1. [file elife-108037-fig5-figsupp1-data1.zip › eLife-108037R1-Figure5-figure supplement 1/Figure5-figure supplement 1C-p-AKT-3.tif]

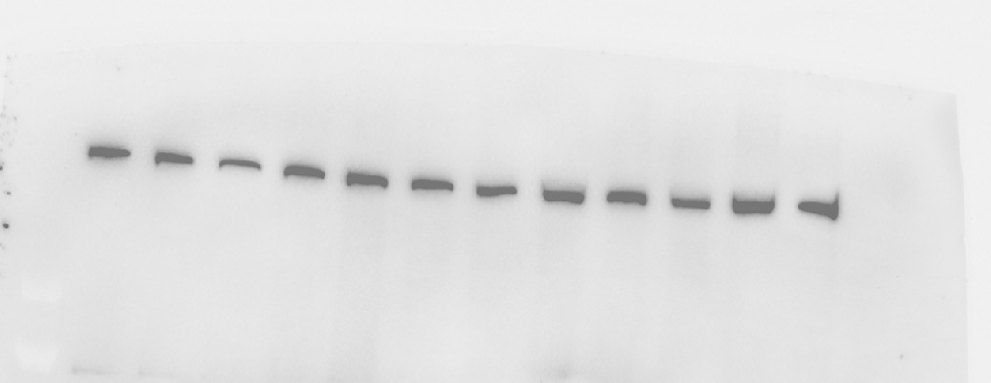

Supplement: Figure 5—figure supplement 1—source data 1. [file elife-108037-fig5-figsupp1-data1.zip › eLife-108037R1-Figure5-figure supplement 1/Figure5-figure supplement 1C-p-mTOR(S2448)-1.tif]

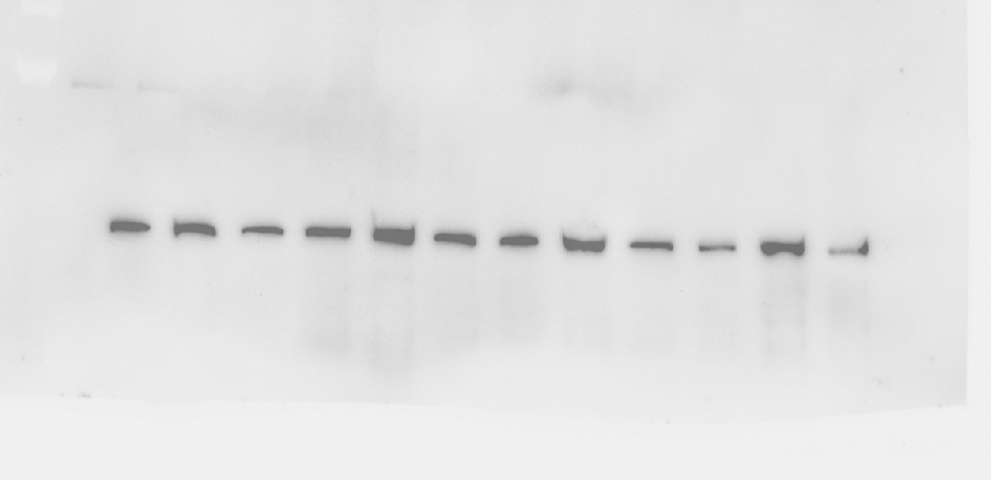

Supplement: Figure 5—figure supplement 1—source data 1. [file elife-108037-fig5-figsupp1-data1.zip › eLife-108037R1-Figure5-figure supplement 1/Figure5-figure supplement 1C-p-mTOR(S2448)-2.tif]

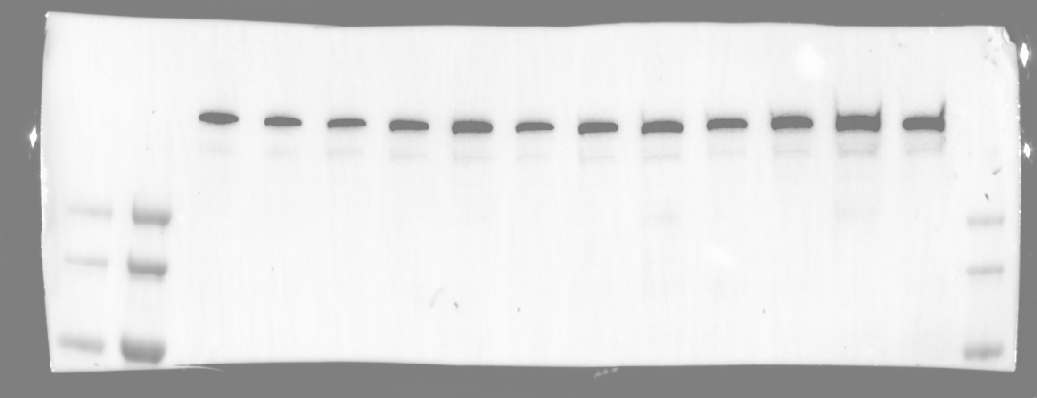

Supplement: Figure 5—figure supplement 1—source data 1. [file elife-108037-fig5-figsupp1-data1.zip › eLife-108037R1-Figure5-figure supplement 1/Figure5-figure supplement 1C-p-mTOR(S2448)-3.tif]

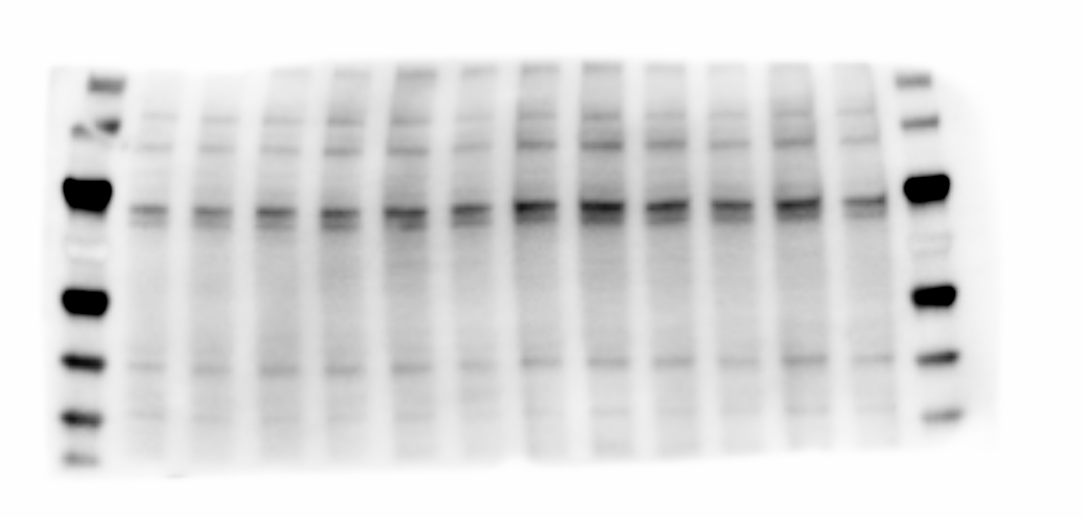

Supplement: Figure 5—figure supplement 1—source data 1. [file elife-108037-fig5-figsupp1-data1.zip › eLife-108037R1-Figure5-figure supplement 1/Figure5-figure supplement 1C-p-p70(S6K)-1.tif]

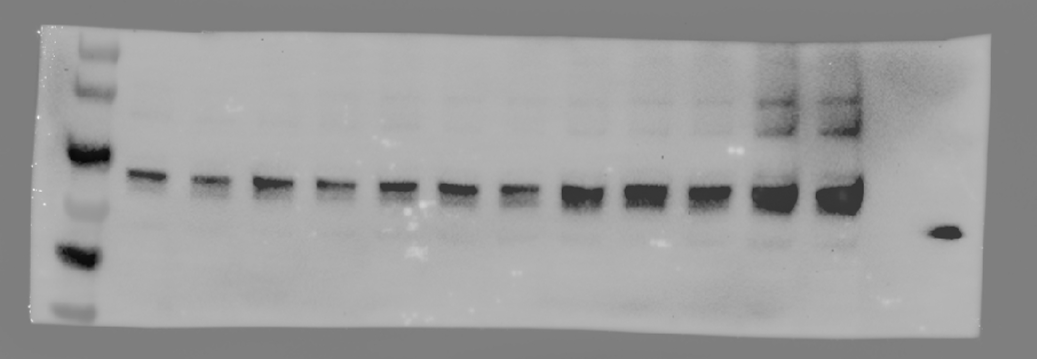

Supplement: Figure 5—figure supplement 1—source data 1. [file elife-108037-fig5-figsupp1-data1.zip › eLife-108037R1-Figure5-figure supplement 1/Figure5-figure supplement 1C-p-p70(S6K)-2.tif]

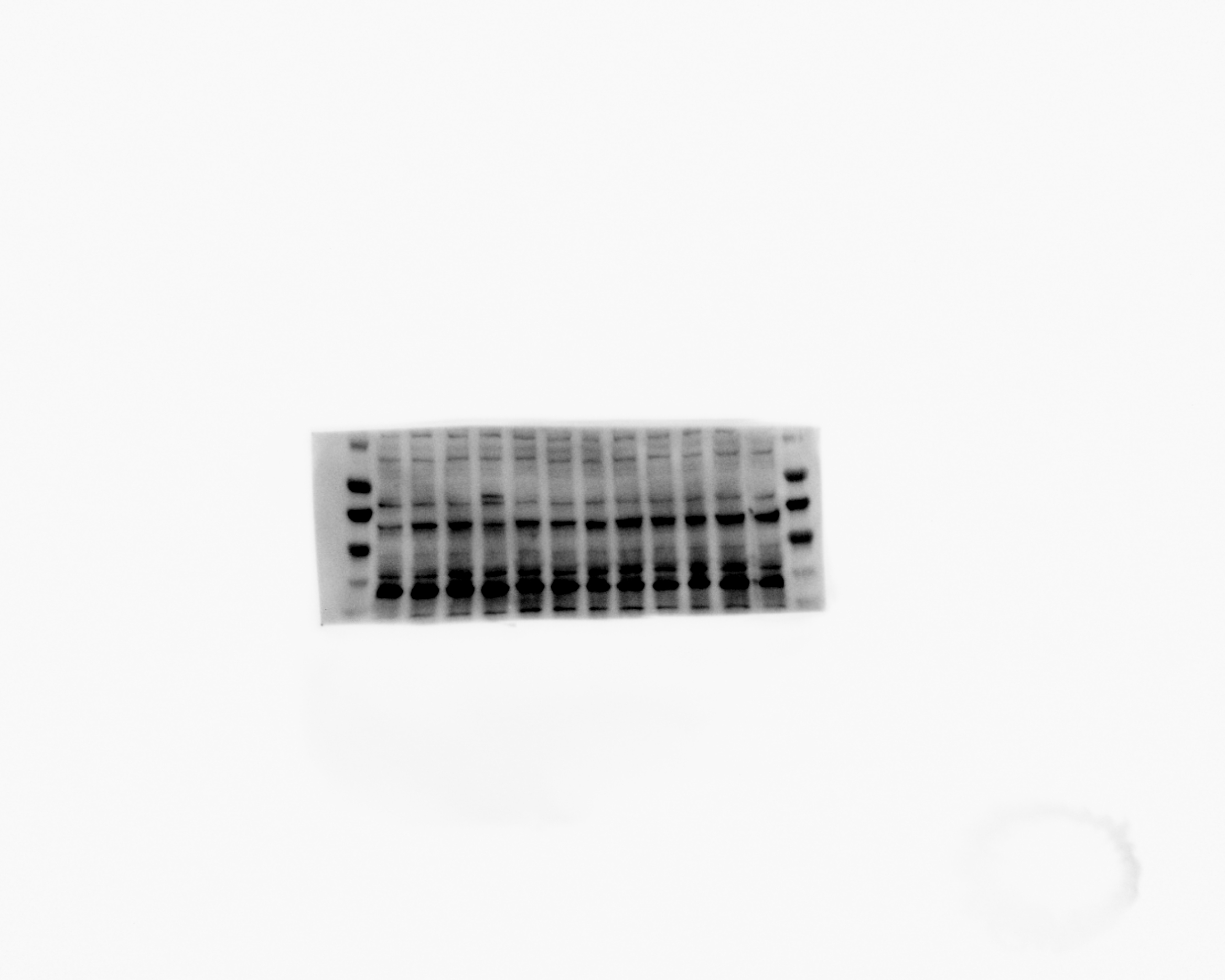

Supplement: Figure 5—figure supplement 1—source data 1. [file elife-108037-fig5-figsupp1-data1.zip › eLife-108037R1-Figure5-figure supplement 1/Figure5-figure supplement 1C-p-p70(S6K)-3.tiff]

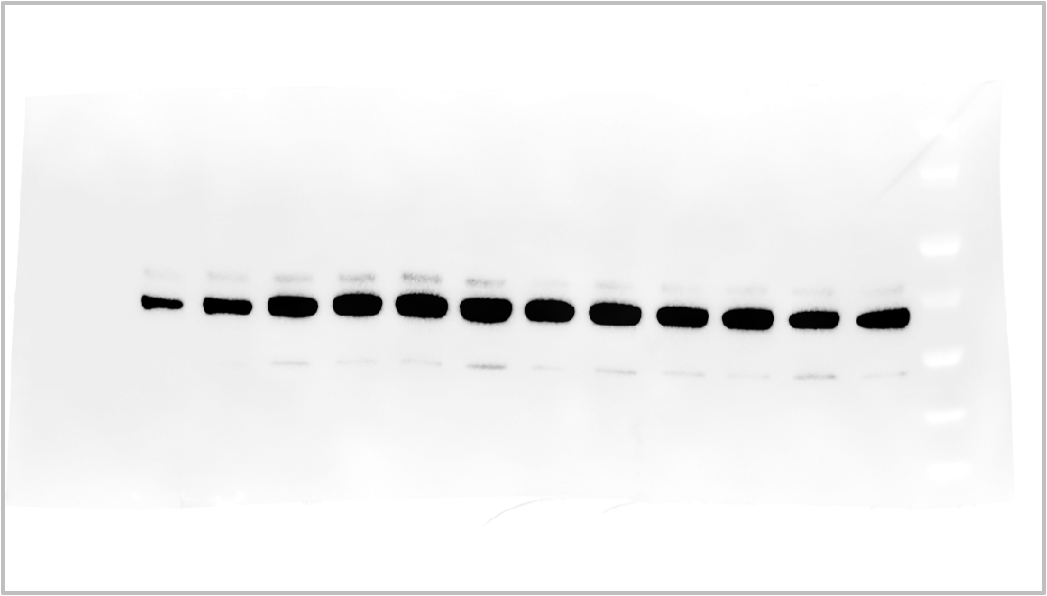

Supplement: Figure 5—figure supplement 1—source data 1. [file elife-108037-fig5-figsupp1-data1.zip › eLife-108037R1-Figure5-figure supplement 1/Figure5-figure supplement 1C-p70(S6K).tif]

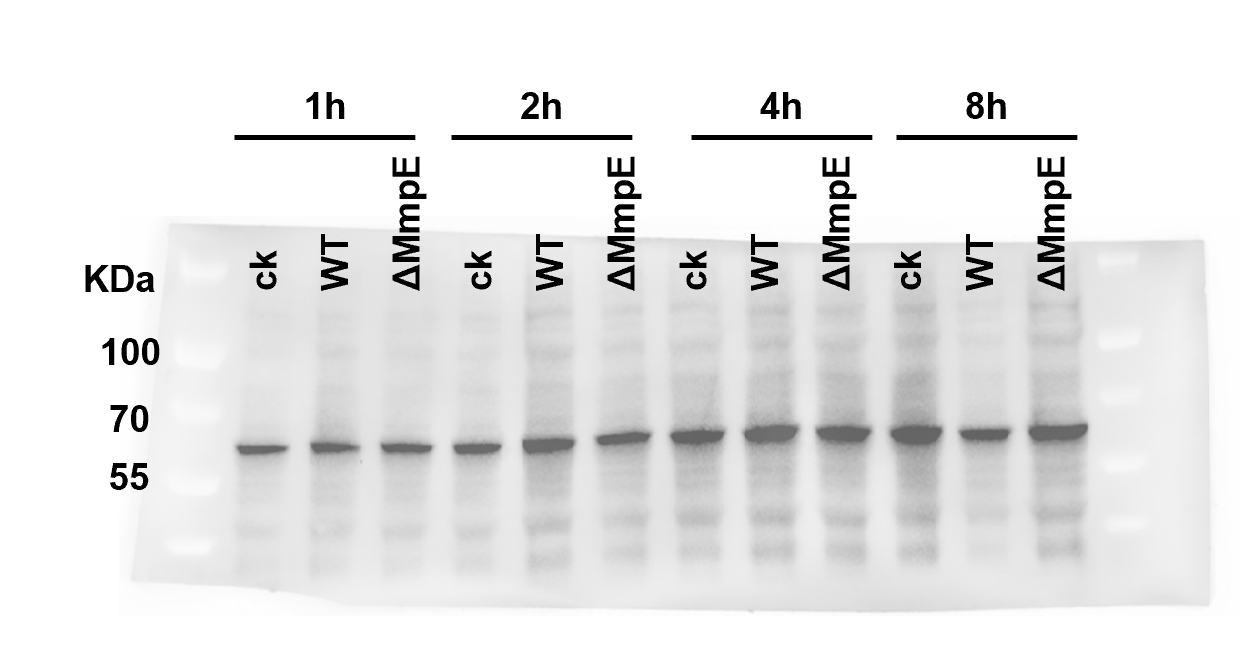

Supplement: Figure 5—figure supplement 1—source data 2. [file elife-108037-fig5-figsupp1-data2.zip › eLife-108037R1-Figure5-figure supplement 1 and notes/Figure5-figure supplement 1C-AKT.tif]

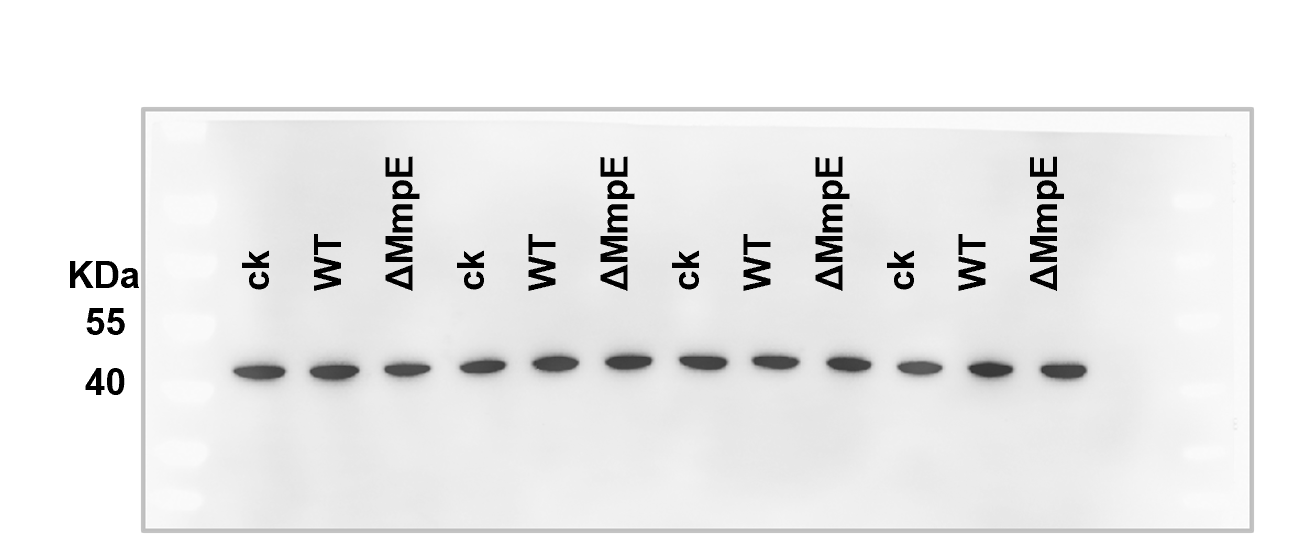

Supplement: Figure 5—figure supplement 1—source data 2. [file elife-108037-fig5-figsupp1-data2.zip › eLife-108037R1-Figure5-figure supplement 1 and notes/Figure5-figure supplement 1C-b-actin.tif]

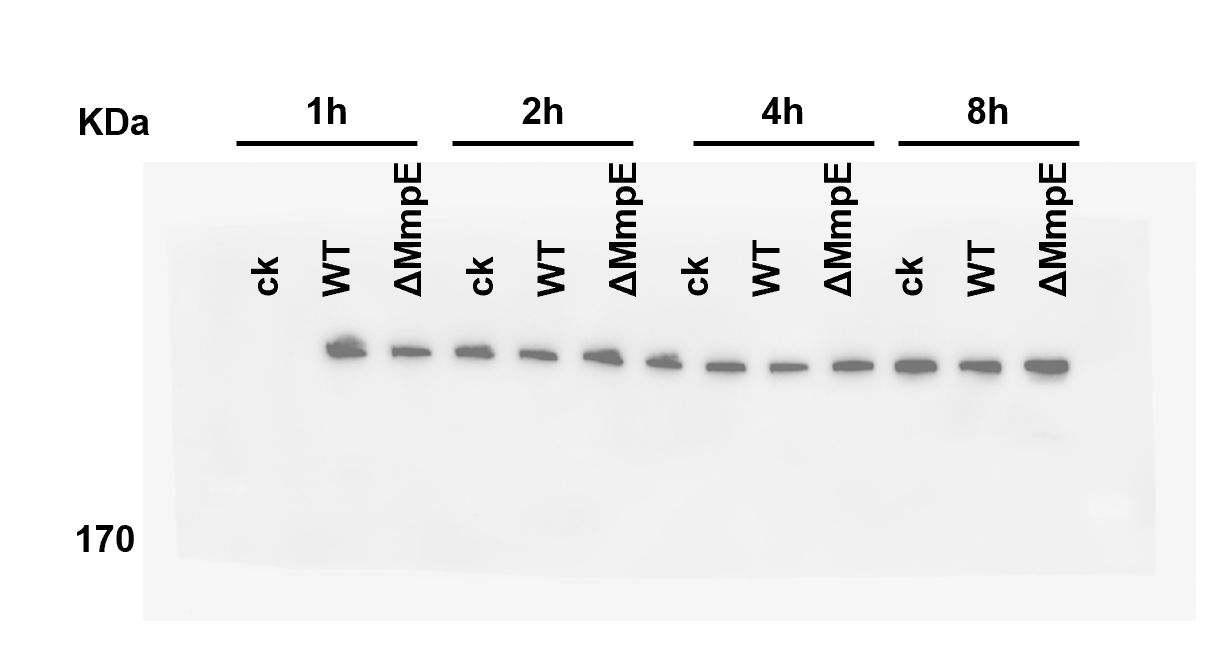

Supplement: Figure 5—figure supplement 1—source data 2. [file elife-108037-fig5-figsupp1-data2.zip › eLife-108037R1-Figure5-figure supplement 1 and notes/Figure5-figure supplement 1C-mTOR.tif]

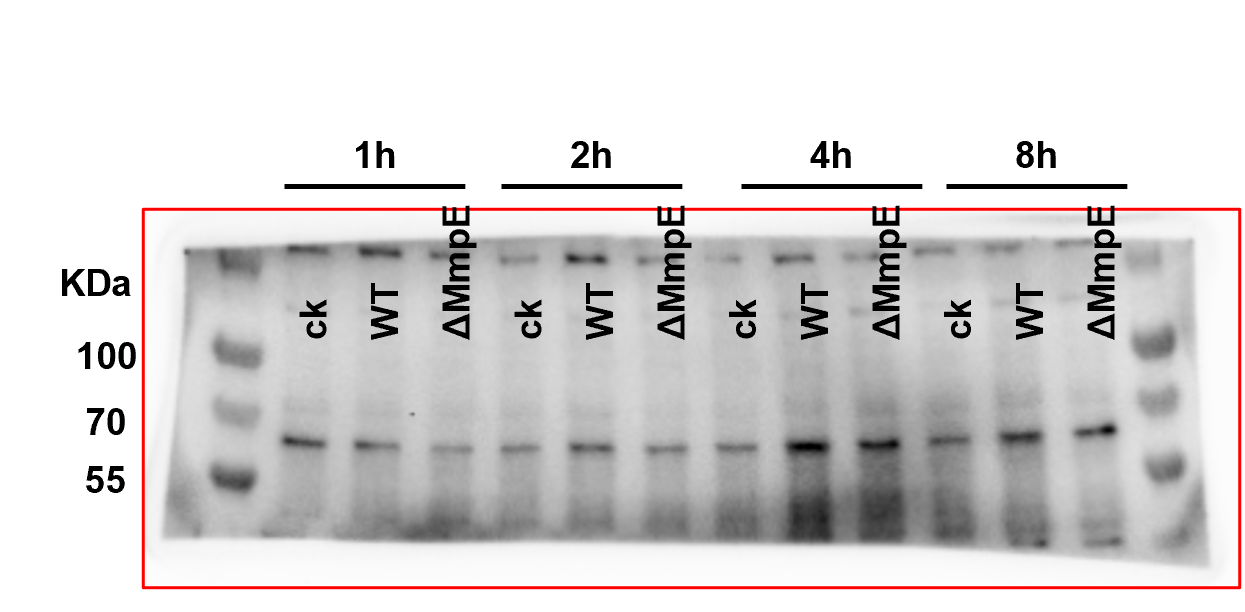

Supplement: Figure 5—figure supplement 1—source data 2. [file elife-108037-fig5-figsupp1-data2.zip › eLife-108037R1-Figure5-figure supplement 1 and notes/Figure5-figure supplement 1C-p-AKT-1.tif]

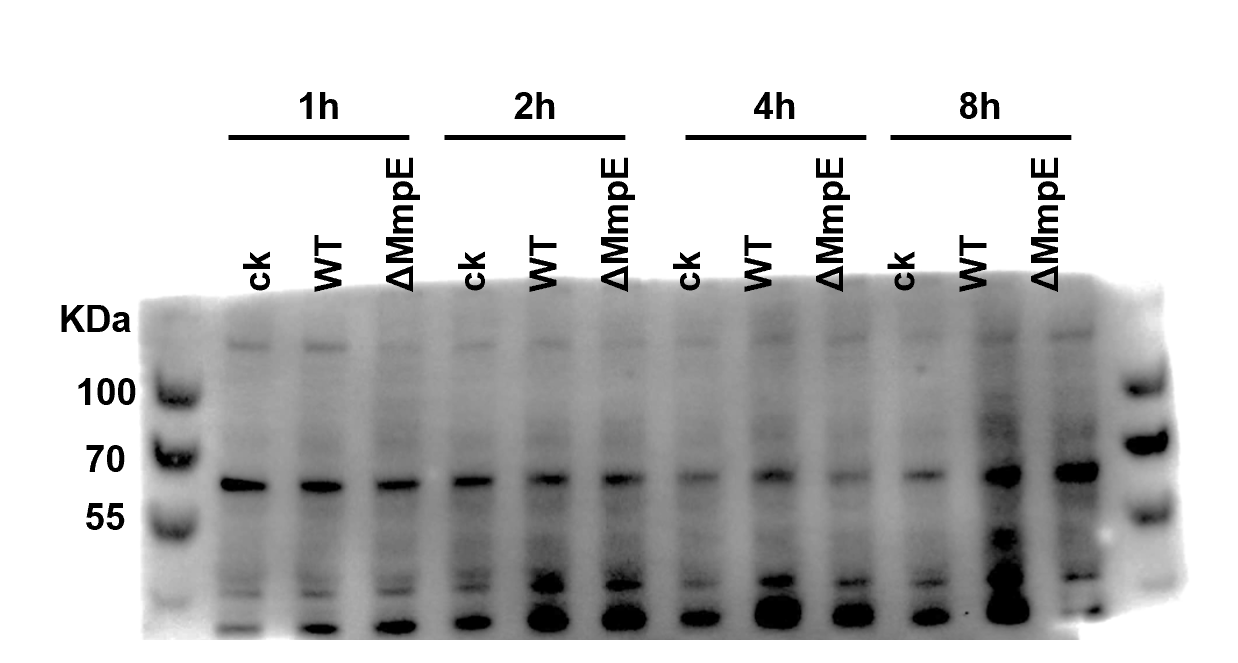

Supplement: Figure 5—figure supplement 1—source data 2. [file elife-108037-fig5-figsupp1-data2.zip › eLife-108037R1-Figure5-figure supplement 1 and notes/Figure5-figure supplement 1C-p-AKT-2.tif]

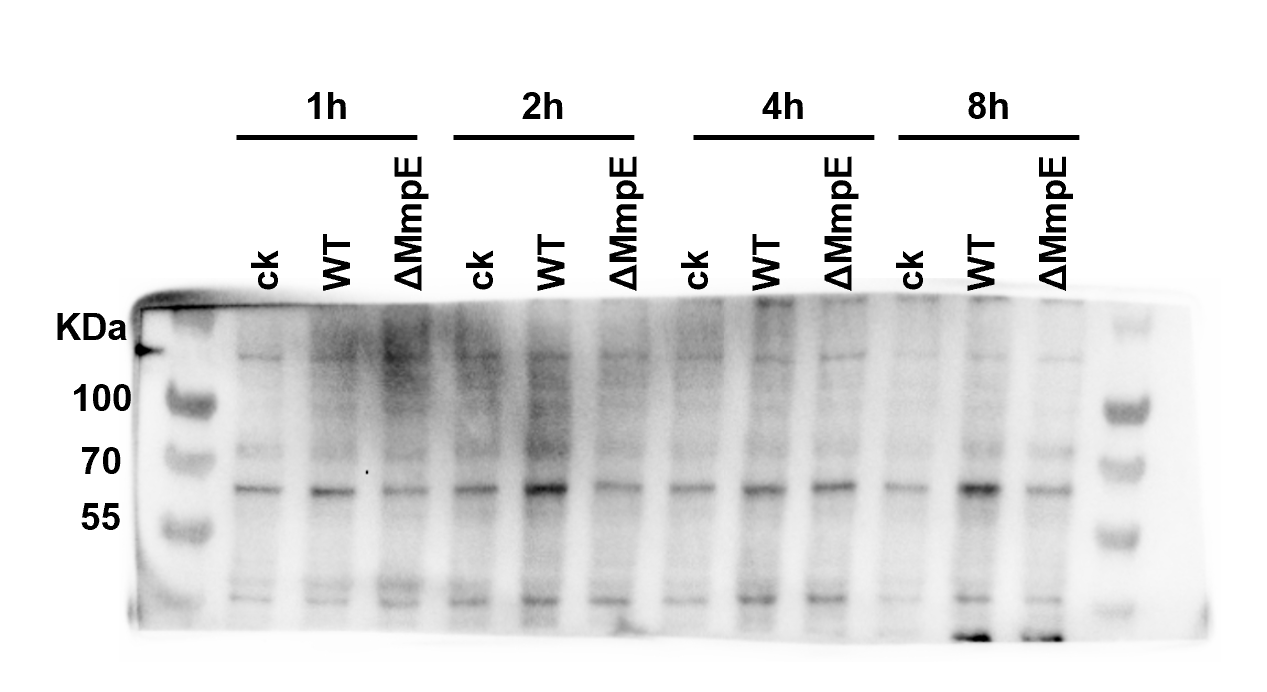

Supplement: Figure 5—figure supplement 1—source data 2. [file elife-108037-fig5-figsupp1-data2.zip › eLife-108037R1-Figure5-figure supplement 1 and notes/Figure5-figure supplement 1C-p-AKT-3.tif]

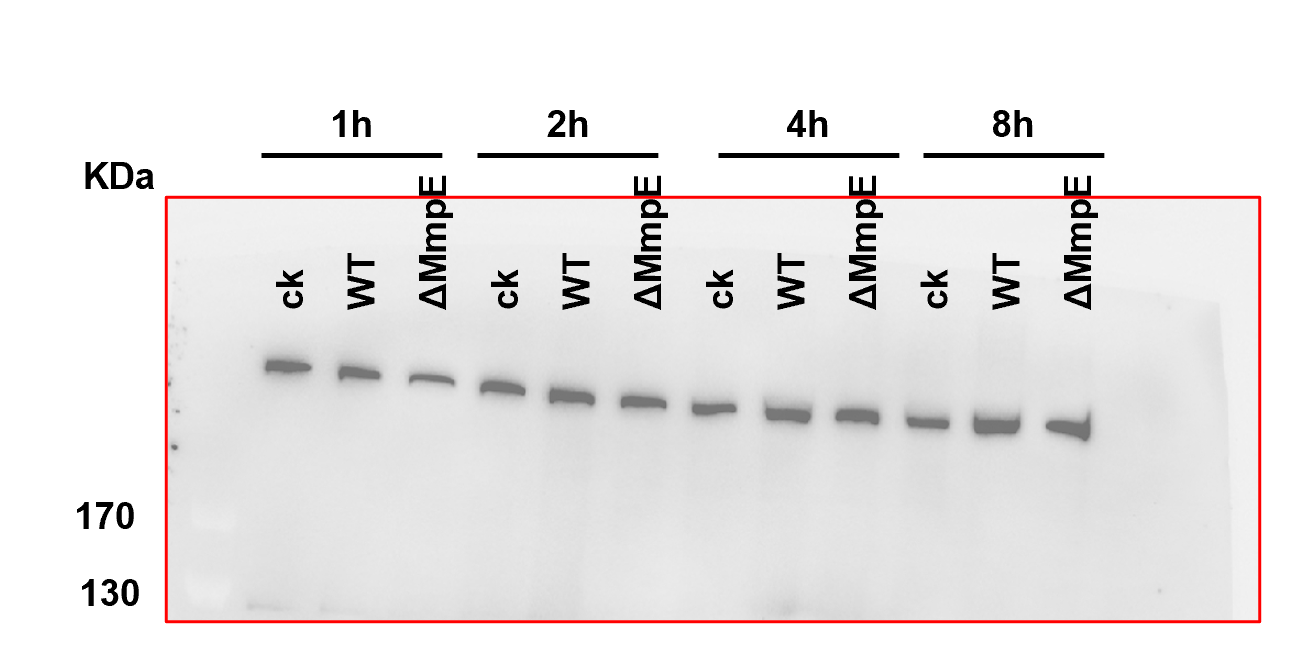

Supplement: Figure 5—figure supplement 1—source data 2. [file elife-108037-fig5-figsupp1-data2.zip › eLife-108037R1-Figure5-figure supplement 1 and notes/Figure5-figure supplement 1C-p-mTOR(S2448)-1.tif]

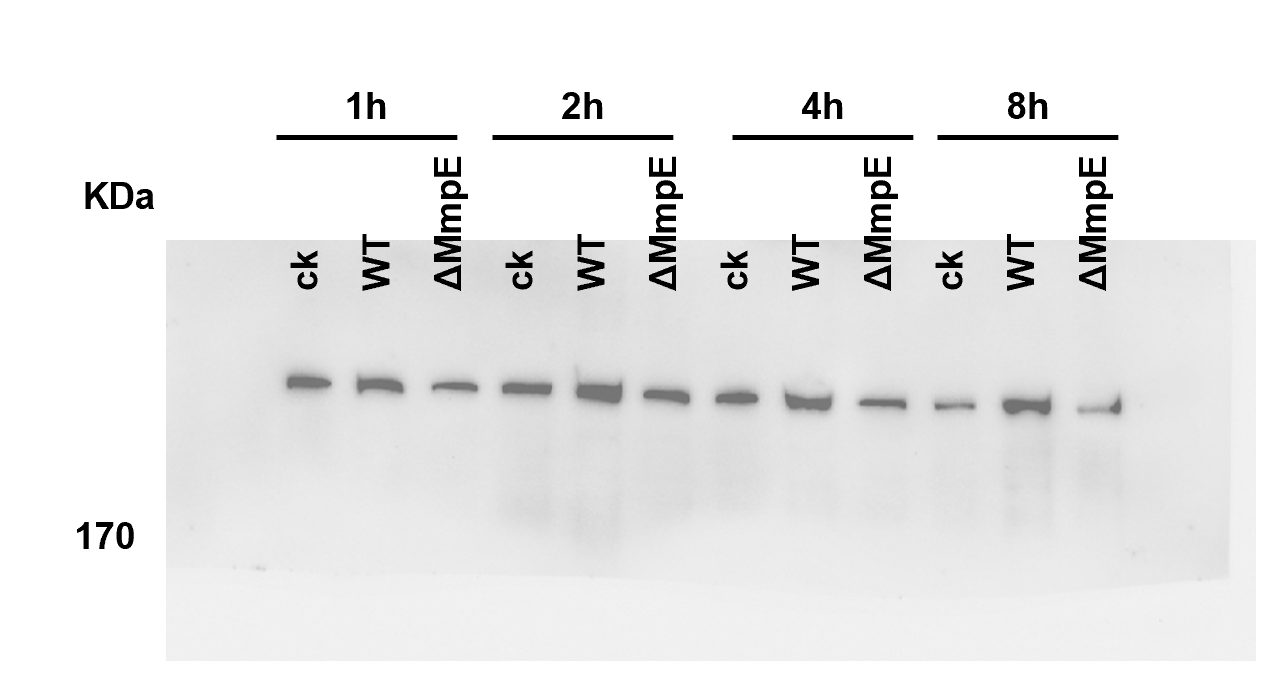

Supplement: Figure 5—figure supplement 1—source data 2. [file elife-108037-fig5-figsupp1-data2.zip › eLife-108037R1-Figure5-figure supplement 1 and notes/Figure5-figure supplement 1C-p-mTOR(S2448)-2.tif]

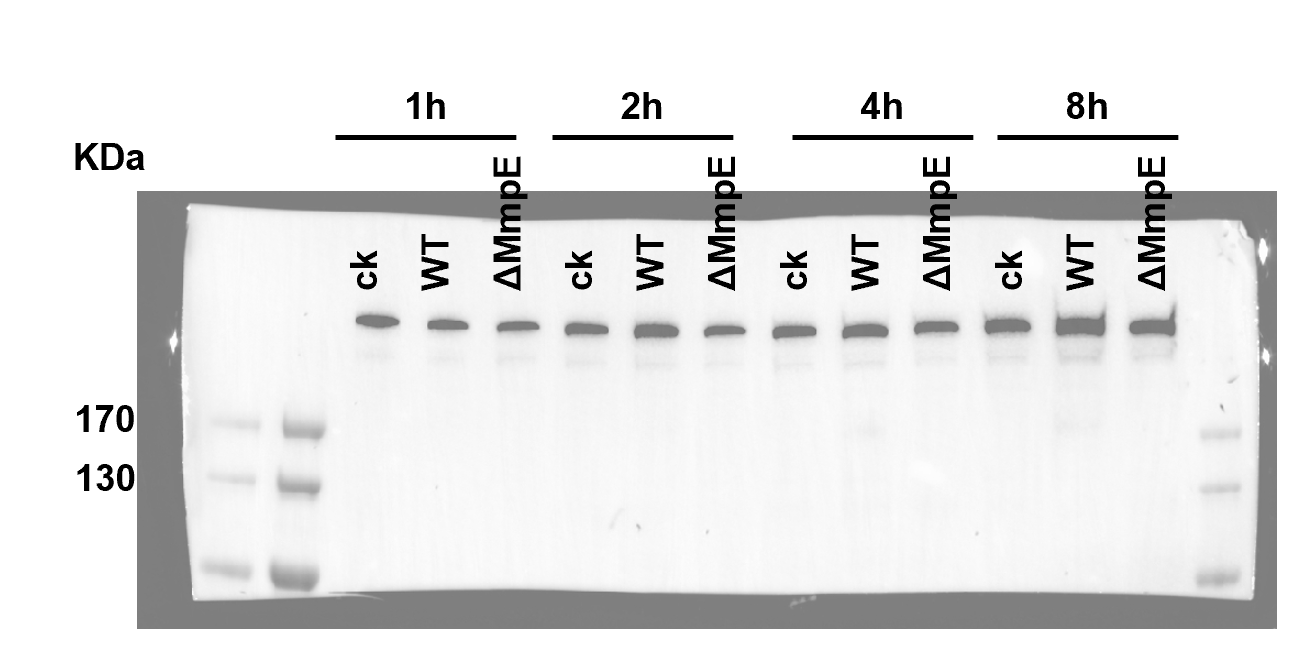

Supplement: Figure 5—figure supplement 1—source data 2. [file elife-108037-fig5-figsupp1-data2.zip › eLife-108037R1-Figure5-figure supplement 1 and notes/Figure5-figure supplement 1C-p-mTOR(S2448)-3.tif]

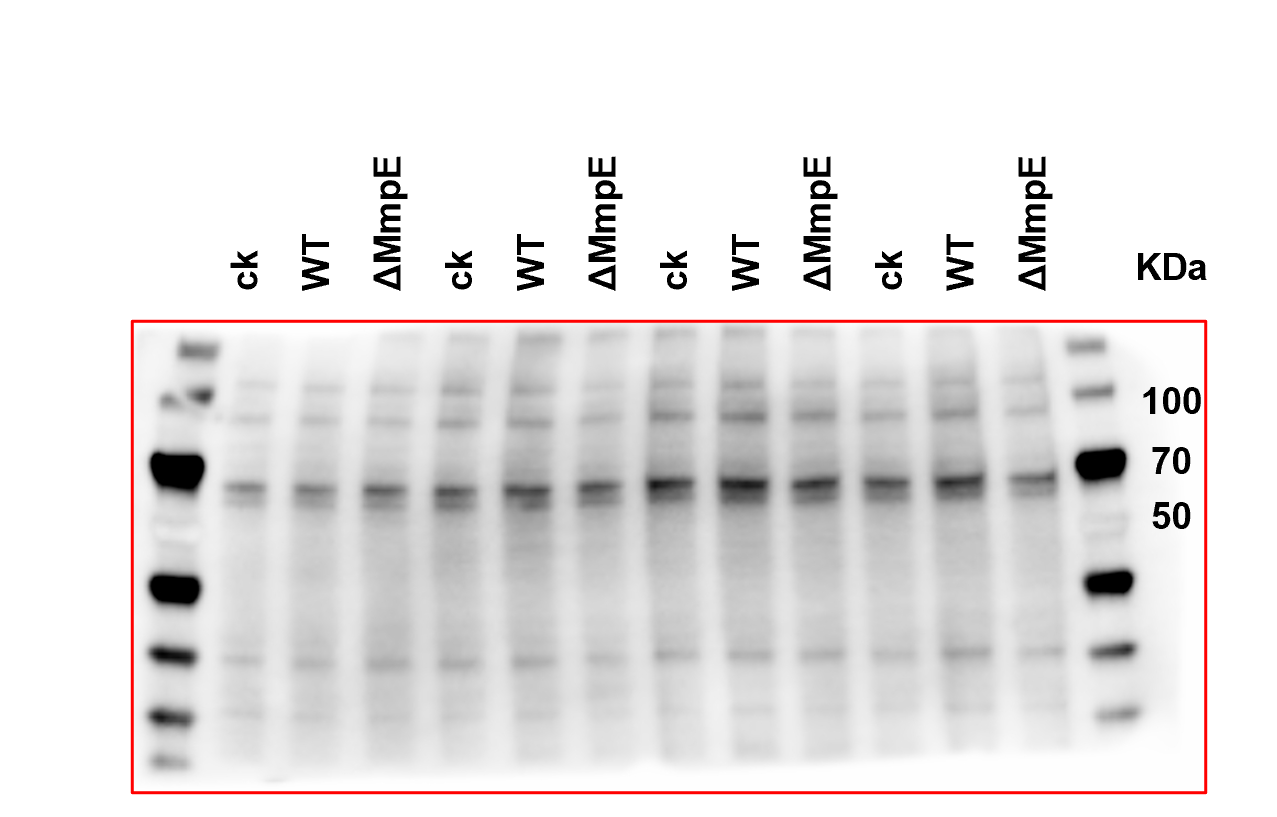

Supplement: Figure 5—figure supplement 1—source data 2. [file elife-108037-fig5-figsupp1-data2.zip › eLife-108037R1-Figure5-figure supplement 1 and notes/Figure5-figure supplement 1C-p-p70(S6K)-1.tif]

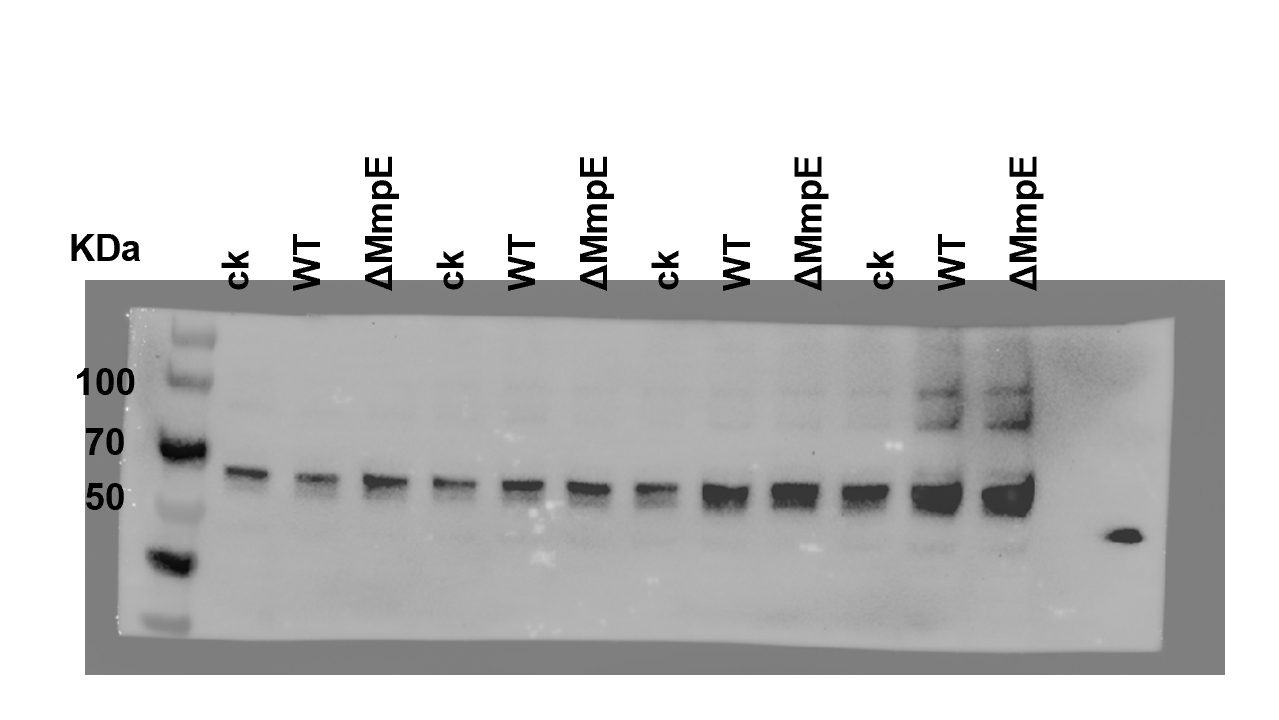

Supplement: Figure 5—figure supplement 1—source data 2. [file elife-108037-fig5-figsupp1-data2.zip › eLife-108037R1-Figure5-figure supplement 1 and notes/Figure5-figure supplement 1C-p-p70(S6K)-2.tif]

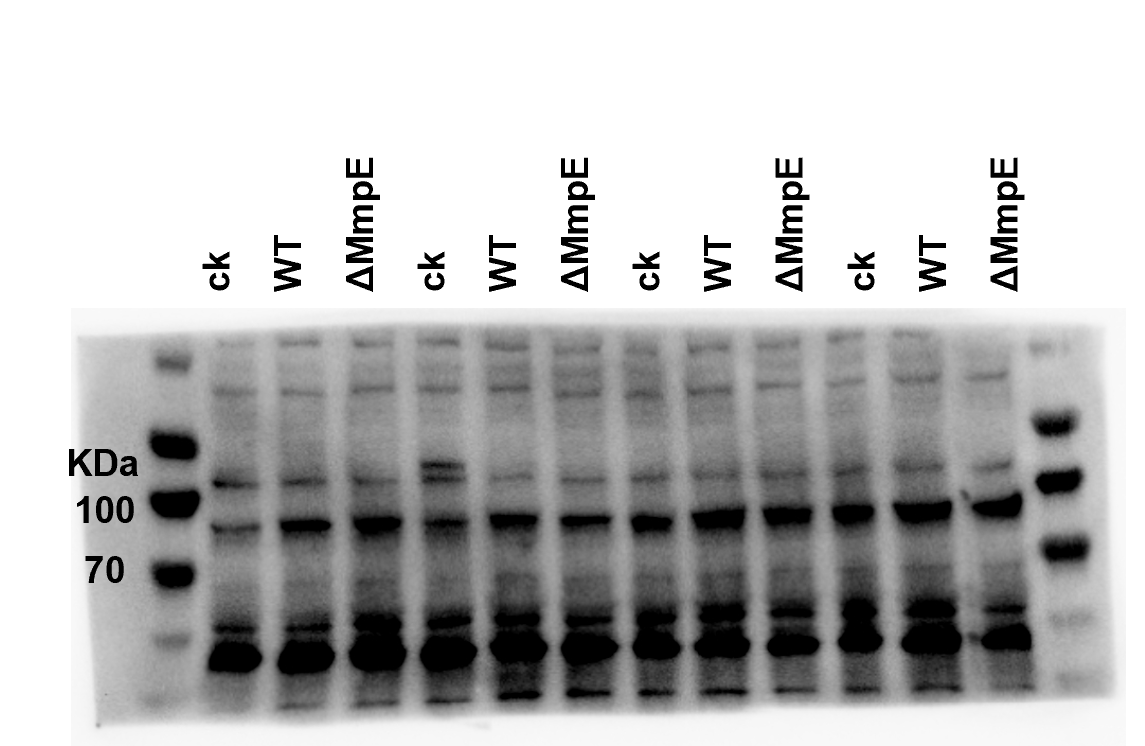

Supplement: Figure 5—figure supplement 1—source data 2. [file elife-108037-fig5-figsupp1-data2.zip › eLife-108037R1-Figure5-figure supplement 1 and notes/Figure5-figure supplement 1C-p-p70(S6K)-3.tif]

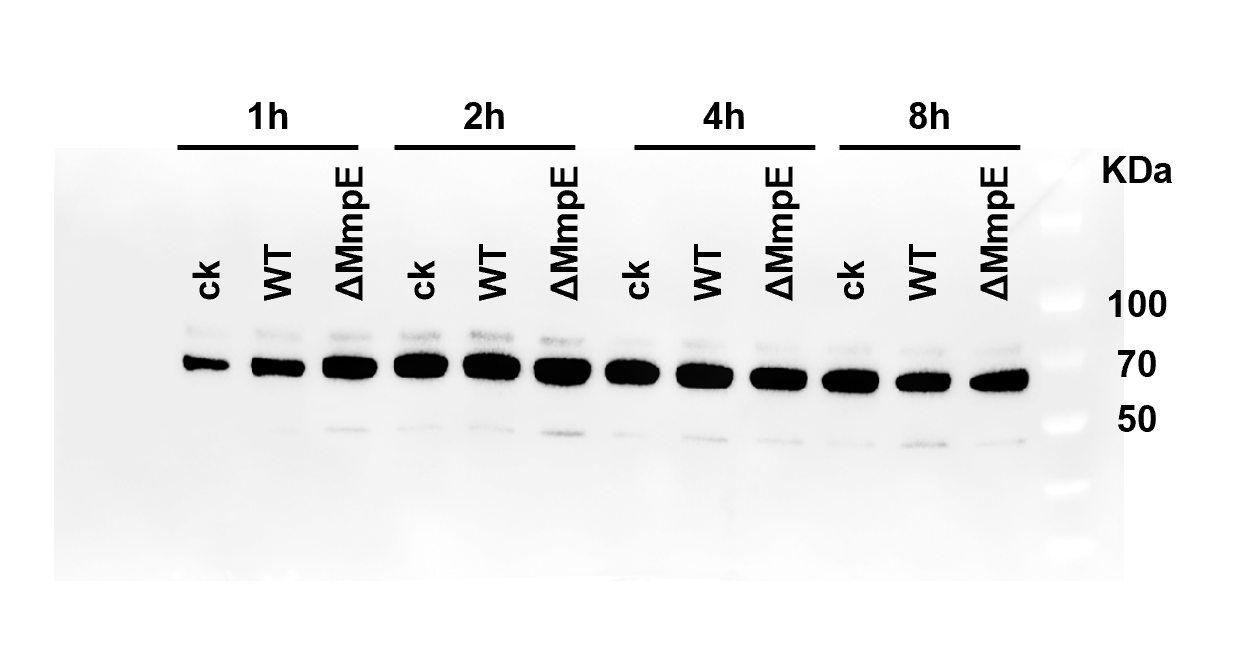

Supplement: Figure 5—figure supplement 1—source data 2. [file elife-108037-fig5-figsupp1-data2.zip › eLife-108037R1-Figure5-figure supplement 1 and notes/Figure5-figure supplement 1C-p70(S6K).tif]

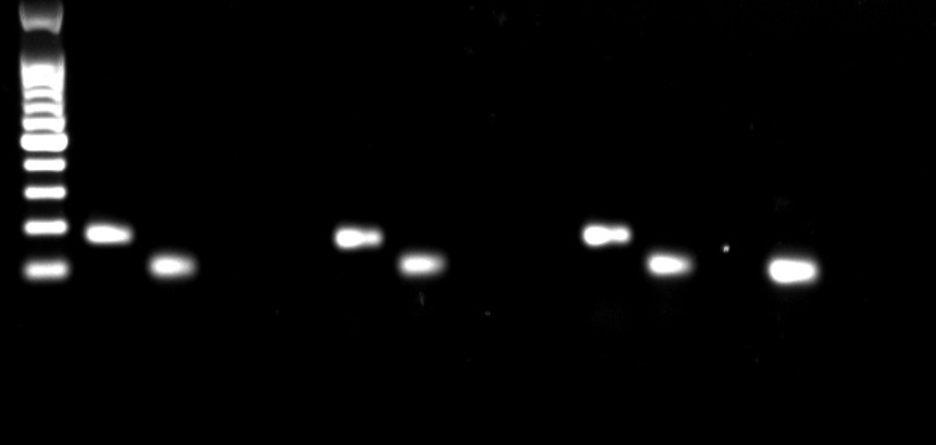

Supplement: Figure 6—source data 1. [file elife-108037-fig6-data1.zip › eLife-108037R1-Figure 6-sourse data/Figure 6D-qPCR.tif]

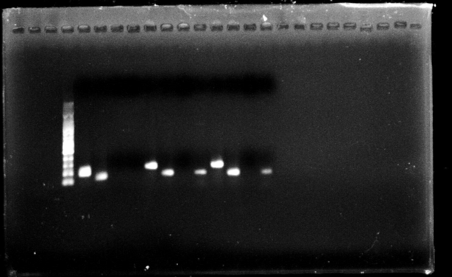

Supplement: Figure 6—source data 1. [file elife-108037-fig6-data1.zip › eLife-108037R1-Figure 6-sourse data/Figure 6F-qPCR.tif]

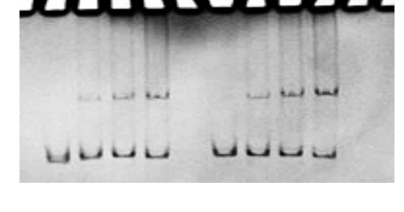

Supplement: Figure 6—source data 1. [file elife-108037-fig6-data1.zip › eLife-108037R1-Figure 6-sourse data/Figure 6H-EMSA-1 and 2.tif]

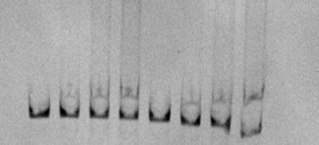

Supplement: Figure 6—source data 1. [file elife-108037-fig6-data1.zip › eLife-108037R1-Figure 6-sourse data/Figure 6H-EMSA-3.tif]

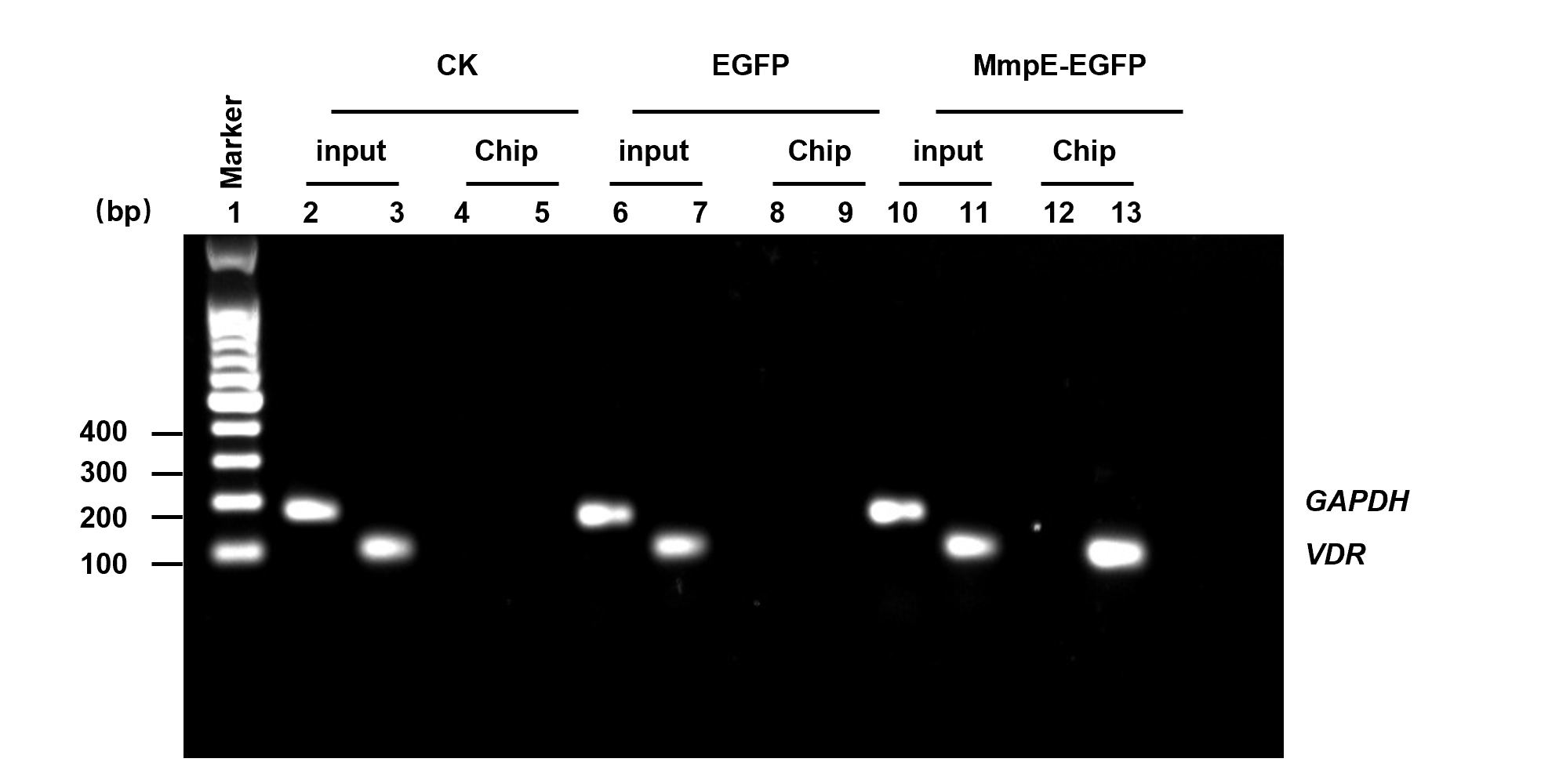

Supplement: Figure 6—source data 2. [file elife-108037-fig6-data2.zip › eLife-108037R1-Figure 6-sourse data and notes/Figure 6D-qPCR.tif]

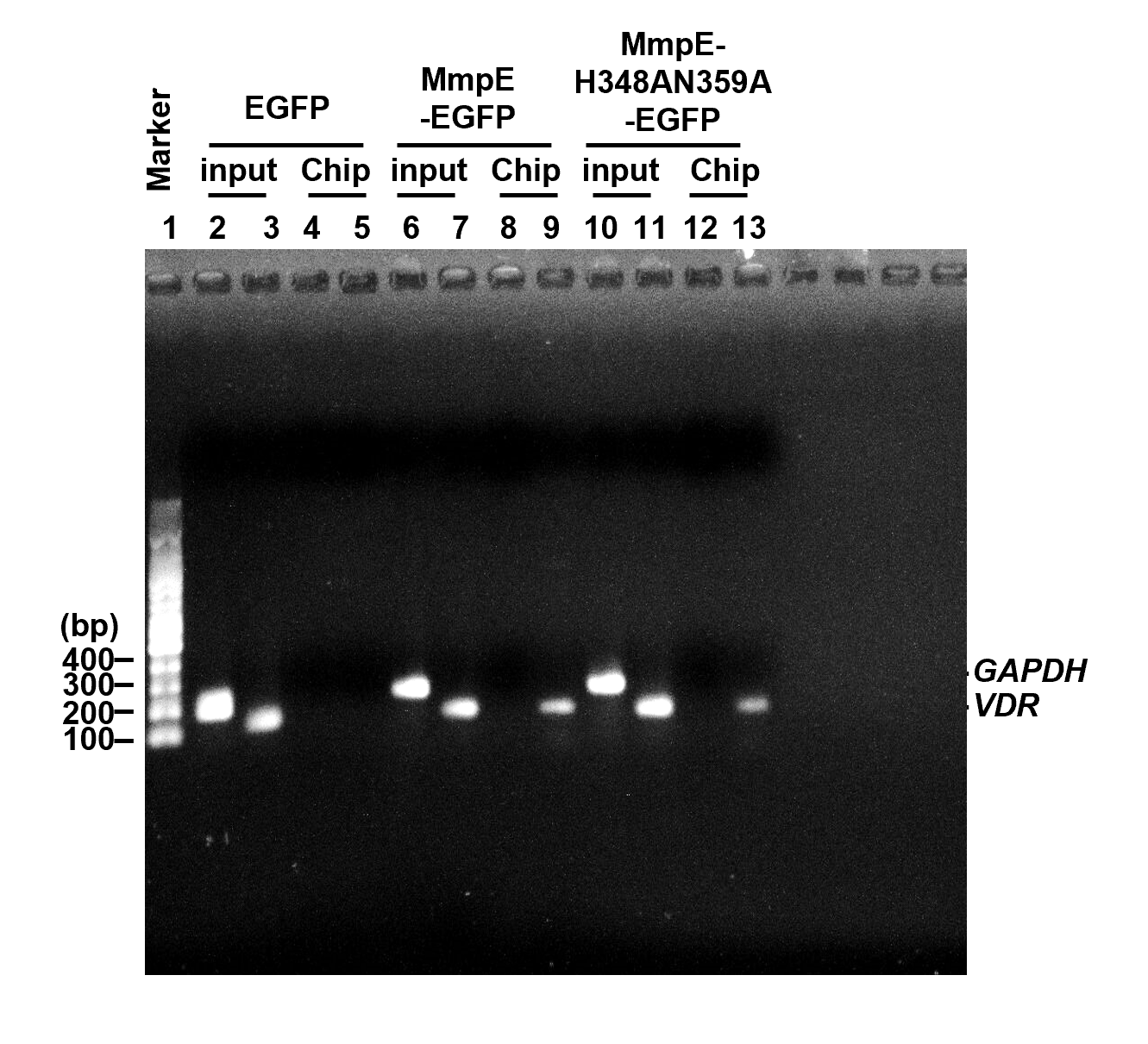

Supplement: Figure 6—source data 2. [file elife-108037-fig6-data2.zip › eLife-108037R1-Figure 6-sourse data and notes/Figure 6F-qPCR.tif]

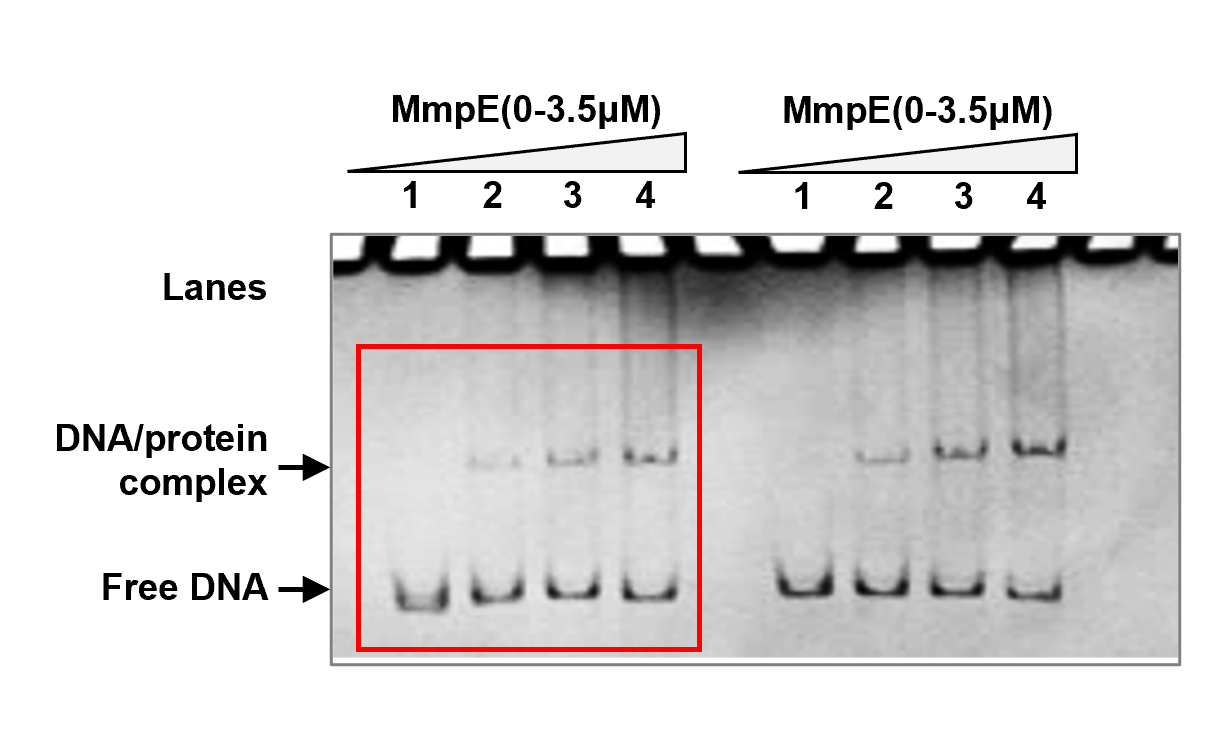

Supplement: Figure 6—source data 2. [file elife-108037-fig6-data2.zip › eLife-108037R1-Figure 6-sourse data and notes/Figure 6H-EMSA-1 and 2.tif]

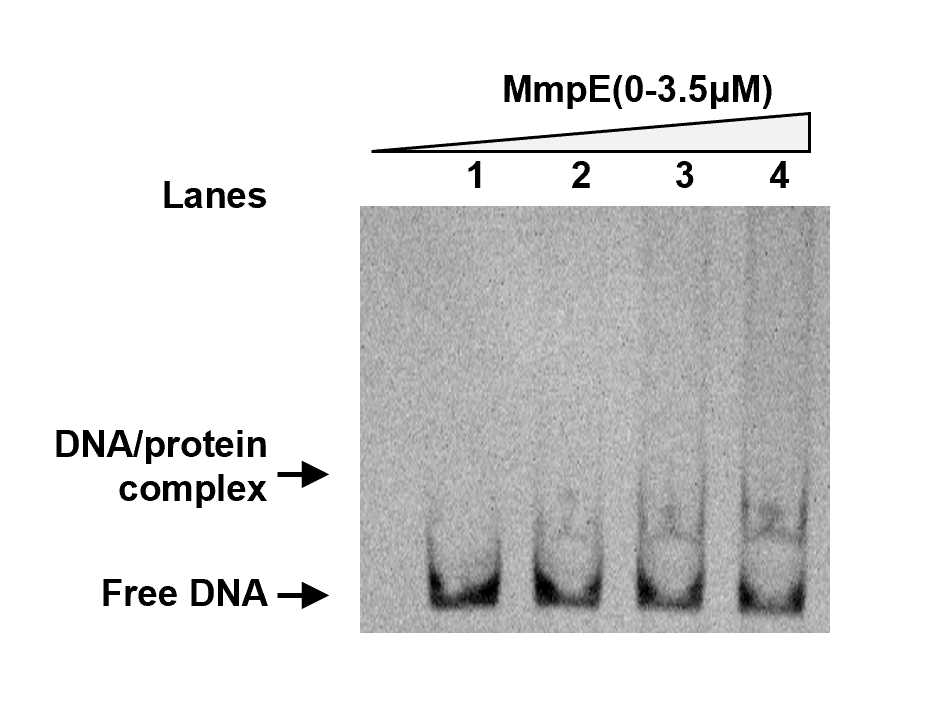

Supplement: Figure 6—source data 2. [file elife-108037-fig6-data2.zip › eLife-108037R1-Figure 6-sourse data and notes/Figure 6H-EMSA-3.tif]
