# Supplementary material for: Mycobacterial metallophosphatase MmpE acts as a nucleomodulin to regulate host gene expression and promote intracellular survival
Source: eLife. 2026 Mar 18;14:RP108037. doi: 10.7554/eLife.108037 (PMC12999176; doi:10.7554/eLife.108037)
Supplement: MDAR checklist [file elife-108037-mdarchecklist1.pdf]

eLife

Materials Design Analysis Reporting (MDAR) Checklist for Authors

The MDAR framework establishes a minimum set of requirements in transparent reporting mainly applicable to studies in the life sciences.

eLife asks authors to **provide detailed information within their article** to facilitate the interpretation and replication of their work. Authors can also upload supporting materials to comply with relevant reporting guidelines for health-related research (see EQUATOR Network), life science research (see the BioSharing Information Resource), or animal research (see the ARRIVE Guidelines and the STRANGE Framework; for details, see *eLife*'s Journal Policies). Where applicable, authors should refer to any relevant reporting standards materials in this form.

For all that apply, please note **where in the article** the information is provided. Please note that we also collect information about data availability and ethics in the submission form.

Materials:

Newly created materials	Indicate where provided: section/figure legend	N/A
The manuscript includes a dedicated "materials availability statement" providing transparent disclosure about availability of newly created materials including details on how materials can be accessed and describing any restrictions on access.		√

Antibodies	Indicate where provided: section/figure legend	N/A
For commercial reagents, provide supplier name, catalogue number and RRID, if available.	See Supplementary File 1 Key resources used in this study	

DNA and RNA sequences	Indicate where provided: section/figure legend	N/A
Short novel DNA or RNA including primers, probes: Sequences should be included or deposited in a public repository.	See Supplementary File7 Primers used in this study	
**Cell materials**	**Indicate where provided: section/figure legend**	**N/A**
Cell lines: Provide species information, strain. Provide accession number in repository OR supplier name, catalog number, clone number, OR RRID.	See Supplementary File 1 Key resources used in this study	
Primary cultures: Provide species, strain, sex of origin, genetic modification status.	See Supplementary File 1 Key resources used in this study; See Supplementary File6 Bacterial strains used in this study	

Experimental animals	Indicate where provided: section/figure legend	N/A
Laboratory animals or Model organisms: Provide species, strain, sex, age, genetic modification status. Provide accession number in repository OR supplier name, catalog number, clone number, OR RRID.	See Supplementary File 1 Key resources used in this study	
Animal observed in or captured from the field: Provide species, sex, and age where possible.		✓

Plants and microbes	Indicate where provided: section/figure legend	N/A
Plants: provide species and strain, ecotype and cultivar where relevant, unique accession number if available, and source (including location for collected wild specimens).		✓
Microbes: provide species and strain, unique accession number if available, and source.	See Supplementary File 1 Key resources used in this study	

Human research participants	Indicate where provided: section/figure legend) or state if these demographics were not collected	N/A
If collected and within the bounds of privacy constraints report on age, sex, gender and ethnicity for all study participants.		✓

Design:

Study protocol	Indicate where provided: section/figure legend	N/A
If the study protocol has been pre-registered, provide DOI. For clinical trials, provide the trial registration number OR cite DOI.		✓

Laboratory protocol	Indicate where provided: section/figure legend	N/A
Provide DOI OR other citation details if detailed step-by-step protocols are available.	See Materials and Methods section in the main text	

Experimental study design (statistics details) *		
**For in vivo studies: State whether and how the following have been done**	**Indicate where provided: section/figure legend. If it could have been done, but was not, write "not done"**	**N/A**
Sample size determination	Each experiment included 6 biological replicates per sample. See Materials and Methods, "Animal experiments" section.	
Randomisation	Randomisation was performed.	
Blinding	A double-blind design was used for experimental conduct and outcome assessment.	
Inclusion/exclusion criteria	Mice aged 6-8 weeks with body weight between 16-18 g were included. See Materials and Methods	

Sample definition and in-laboratory replication	Indicate where provided: section/figure legend	N/A
State number of times the experiment was replicated in the laboratory.	Immunoblot analysis was performed with 3 technical replicates. Phosphatase activity assay, Quantitative real-time PCR, EMSA, Phagosomal Acidification detection, and CFU assay each had 3 biological replicates. Chromatin Immunoprecipitation (ChIP) assays were performed without replicates. See Materials and Methods section for details.	
Define whether data describe technical or biological replicates.	Immunoblot analysis data represent technical replicates. All other assays except ChIP represent biological replicates. ChIP assays were single runs without replicates. See Materials and Methods section.	

Ethics	Indicate where provided: section/submission form	N/A
Studies involving human participants: State details of authority granting ethics approval (IRB or equivalent committee(s), provide reference number for approval.		✓
Studies involving experimental animals: State details of authority granting ethics approval (IRB or equivalent committee(s), provide reference number for approval.	All animal experiments were conducted in accordance with protocols approved by the Animal Ethics Committee of Huazhong Agricultural University and carried out under the guidelines of the Institutional Animal Care and Use Committee, in compliance with license number SYXK (Hubei) 2020-0084.	
Studies involving specimen and field samples: State if relevant permits obtained, provide details of authority approving study; if none were required, explain why.		✓

Dual Use Research of Concern (DURC)	Indicate where provided: section/submission form	N/A
If study is subject to dual use research of concern regulations, state the authority granting approval and reference number for the regulatory approval.		✓

Analysis:

Attrition	Indicate where provided: section/figure legend	N/A
Describe whether exclusion criteria were pre-established. Report if sample or data points were omitted from analysis. If yes, report if this was due to attrition or intentional exclusion and provide justification.		✓
**Statistics**	**Indicate where provided: section/figure legend**	**N/A**
Describe statistical tests used and justify choice of tests.	Data are presented as mean ± standard deviation (SD). Statistical comparisons were performed using two-tailed unpaired Student’s t-tests or two-way ANOVA. A P-value < 0.05 was considered significant, with significance levels indicated as: *P < 0.05, **P < 0.01, ***P < 0.001.	

Data availability	Indicate where provided: section/submission form	N/A
For newly created and reused datasets, the manuscript includes a data availability statement that provides details for access (or notes restrictions on access).	See Data Availability Statement in the main manuscript.	
When newly created datasets are publicly available, provide accession number in repository OR DOI and licensing details where available.	CUT&Tag dataset: GEO accession number GSE312934; RNA-seq dataset: GEO accession number GSE312039.	
qIf reused data is publicly available provide accession number in repository OR DOI, OR URL, OR citation.		✓

Code availability	Indicate where provided: section/figure legend	N/A
For any computer code/software/mathematical algorithms essential for replicating the main findings of the study, whether newly generated or re-used, the manuscript includes a data availability statement that provides details for access or notes restrictions.		✓
Where newly generated code is publicly available, provide accession number in repository, OR DOI OR URL and licensing details where available. State any restrictions on code availability or accessibility.		✓
If reused code is publicly available provide accession number in repository OR DOI OR URL, OR citation.		✓

Reporting:

The MDAR framework recommends adoption of discipline-specific guidelines, established and endorsed through community initiatives.

Adherence to community standards	Indicate where provided: section/figure legend	N/A
State if relevant guidelines (e.g., ICMJE, MIBBI, ARRIVE, STRANGE) have been followed, and whether a checklist (e.g., CONSORT, PRISMA, ARRIVE) is provided with the manuscript.	See Materials and Methods, “Animal experiments” section	
